# Supplementary material for: Analyses of gut microbiota and plasma bile acids enable stratification of patients for antidiabetic treatment
Source: Nat Commun. 2017 Nov 27;8:1785. doi: 10.1038/s41467-017-01682-2 (PMC5702614; doi:10.1038/s41467-017-01682-2)
Supplement: Supplementary file 1 — Supplementary Information [file 41467_2017_1682_MOESM1_ESM.pdf]

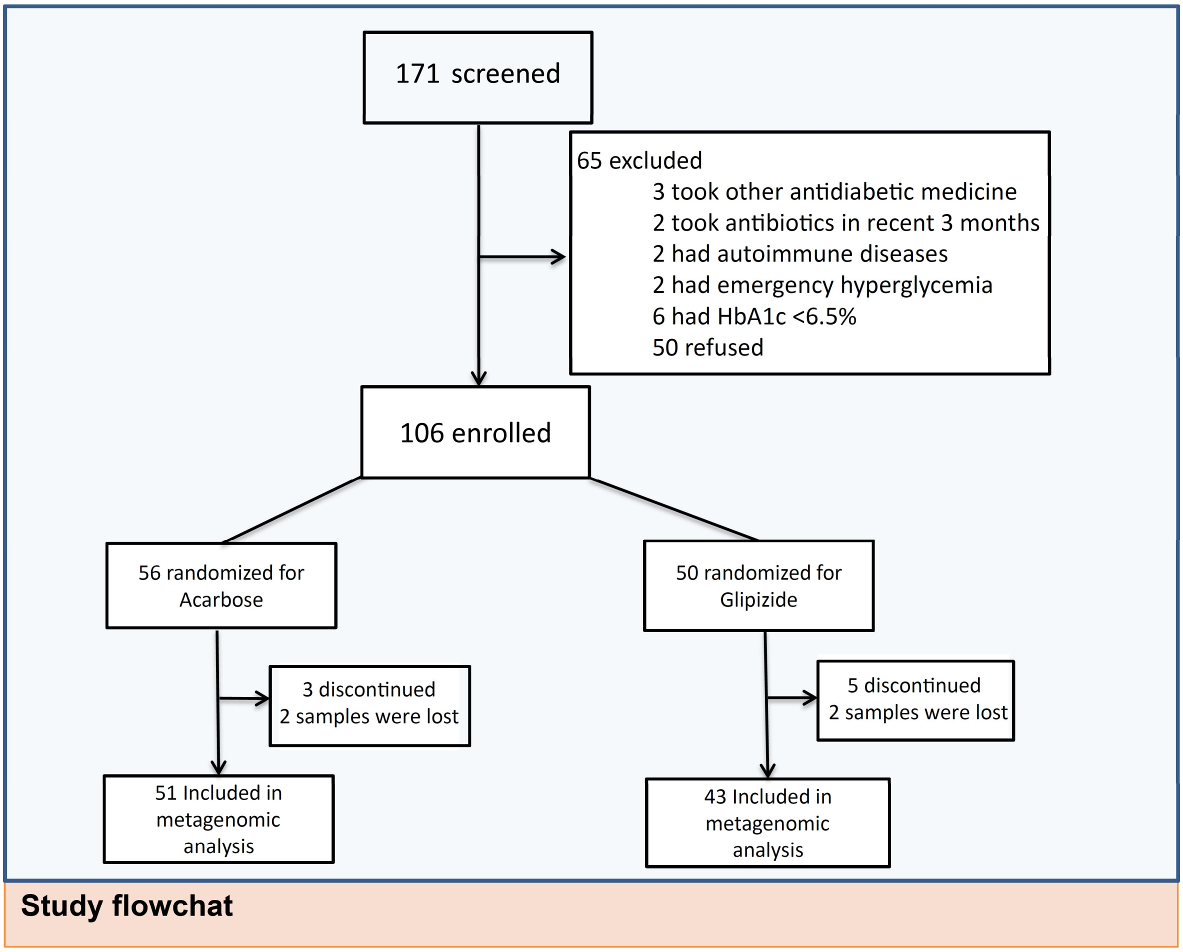

Supplementary Figure 1. Flow chart of the clinical trial

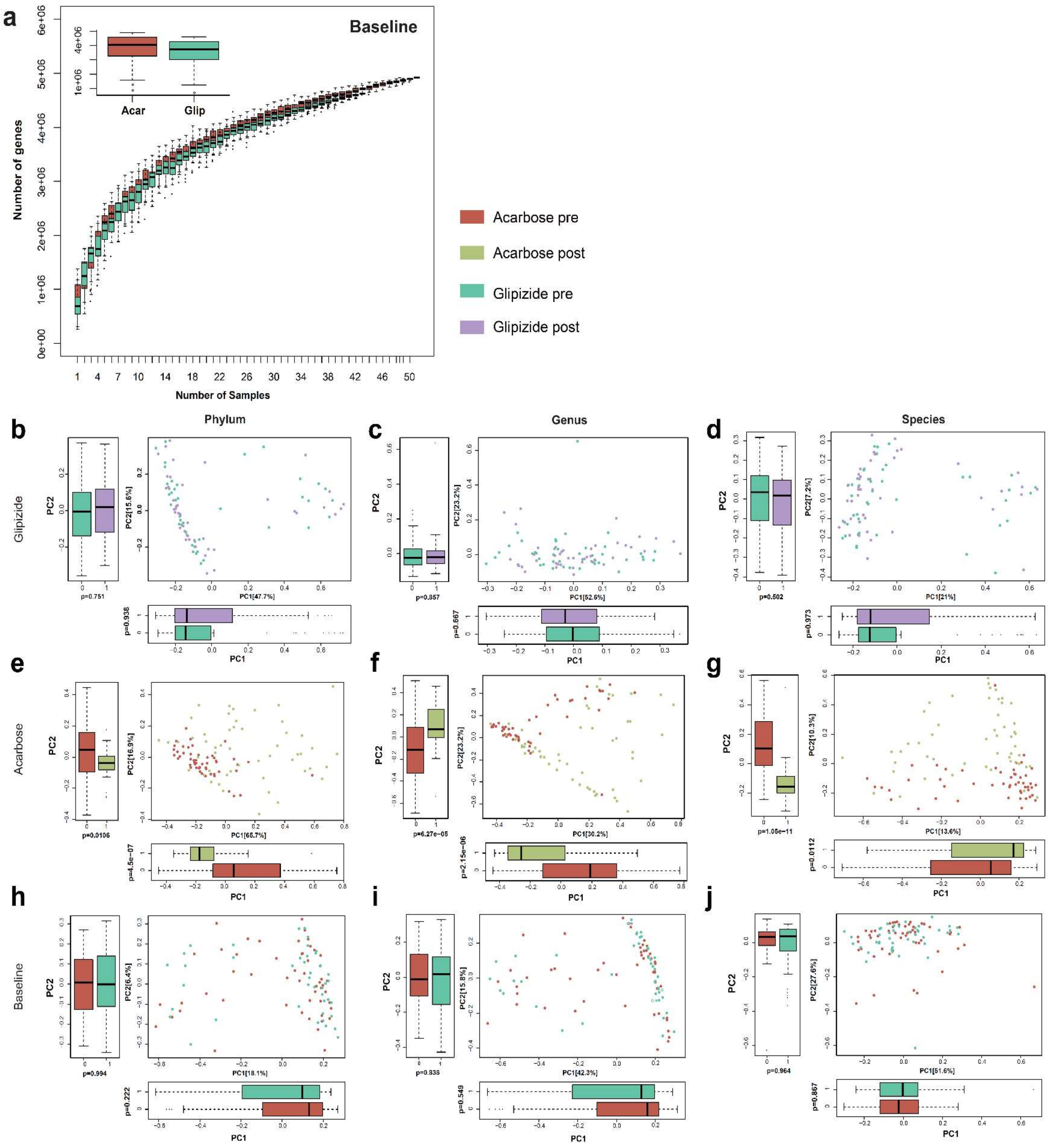
a
Baseline
Acar
Glip
Number of genes
Number of Samples
Acarbose pre
Acarbose post
Glipizide pre
Glipizide post
b
c
d
Phylum
Genus
Species
Glipizide
PC2
PC1
p=0.751
PC2[15.6%]
PC1[47.7%]
p=0.938
p=0.857
PC2[23.2%]
PC1[52.6%]
p=0.667
p=0.502
PC2[7.2%]
PC1[21%]
p=0.973
e
f
g
Acarbose
p=0.0106
PC2[16.9%]
PC1[65.7%]
p=4.5e-07
p=6.27e-05
PC2[23.2%]
PC1[30.2%]
p=2.15e-06
p=1.06e-11
PC2[10.3%]
PC1[13.6%]
p=0.0112
h
i
j
Baseline
p=0.994
PC2[6.4%]
PC1[18.1%]
p=0.222
p=0.838
PC2[15.8%]
PC1[42.3%]
p=0.549
p=0.964
PC2[27.6%]
PC1[51.6%]
p=0.867

Supplementary Figure 2. Metagenomic analysis comparing the two therapeutic arms before and after treatment. (**a**) Rarefaction curves of gut microbial gene content at baseline in both treatment arms. The number of genes in each group was calculated after 100 random samplings with replacement. Plotted boxes are interquartile ranges. Dark lines in the boxes represent medians, the lowest and highest values within 1.5 times IQR from the first and third quartiles (whiskers above and below the boxes). Outliers are shown as circles beyond the whiskers. PCA analysis of taxonomic differences based on profiles at the phylum **(b, e,** and **h)**, genus **(c, f,** and **i)** and species **(d, g,** and **j)** levels. PCAs of samples collected to show the effects of Glipizide on taxonomy in **b, c,** and **d**; effects of Acarbose on taxonomy in **e, f,** and **g**; and baseline comparison of both treatment arms in **h, i,** and **j**. The *P* values were calculated based on Wilcoxon rank-sum test. Acarbose n=51, Glipizide n=43.

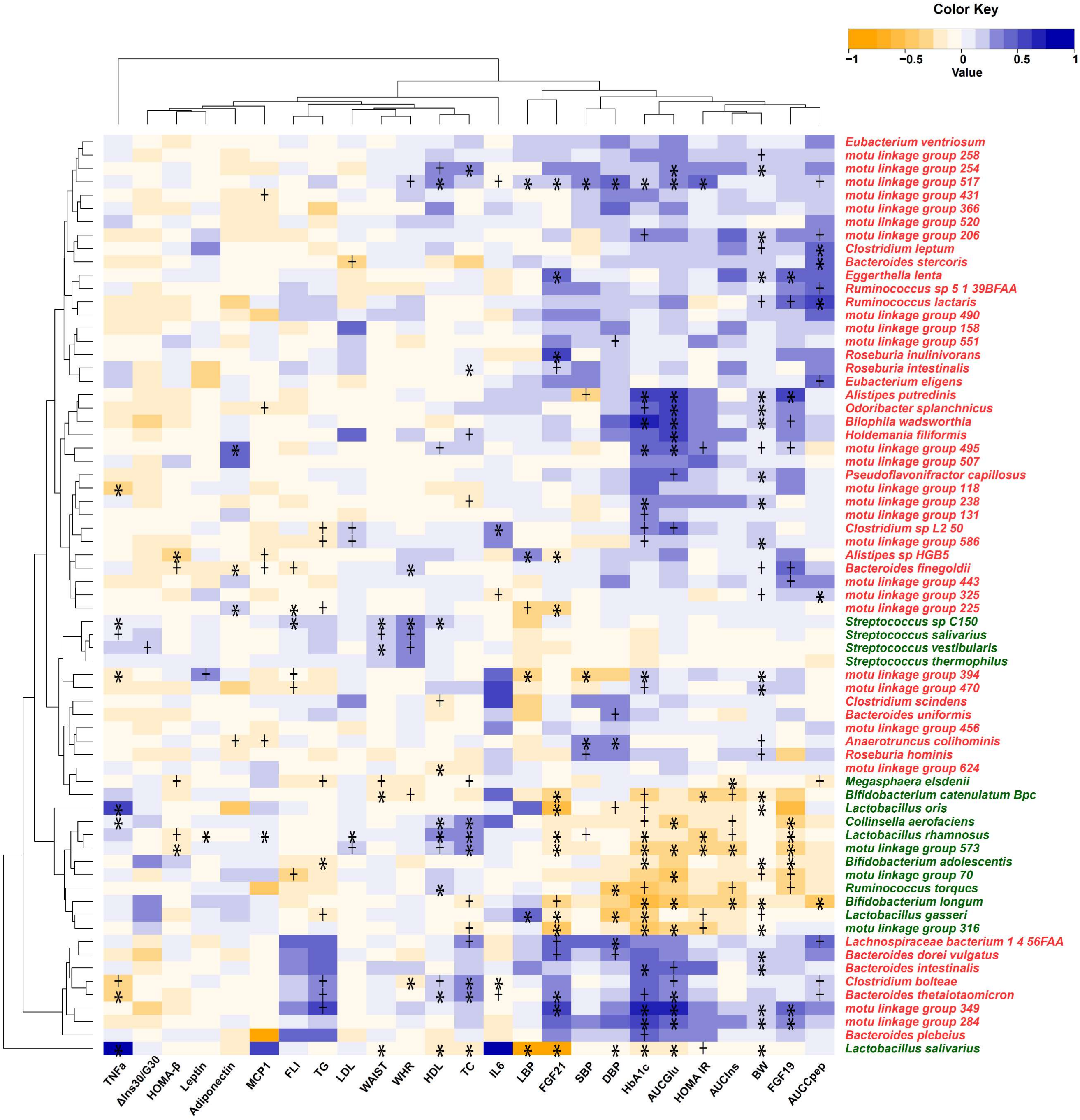

Supplementary Figure 3. Correlation between clinical outcomes and mOTUs that exhibit changes in relative abundance in response to Acarbose treatment. Multivariate longitudinal analysis of the contribution of changes of mOTUs to alterations in clinical outcomes. The colour key indicates the β coefficient value of independent variables. mOTUs in green indicate those

with increased relative abundances after Acarbose treatment; mOTUs in red indicate those with decreased relative abundances after Acarbose treatment; + q<0.05; * q<0.01. BW, body weight; Waist, waist circumference; WHR, waist–hip circumference ratio; HbA1c, glycated haemoglobin; HOMA-IR, homeostasis model assessment of insulin resistance; AUCIns, area under curve value of plasma insulin level during a meal test; AUCCpep, area under curve value of plasma C peptide level during a meal test; AUCGlu, area under curve value of blood glucose level during a meal test; SBP, systolic blood pressure; DBP, diastolic blood pressure; FLI, fatty liver index; LBP, lipopolysaccharides binding protein; ΔIns30/G30= (Ins30-Ins0)/(G30-G0) during a meal test. TC, total cholesterol; LDL, low density of lipoprotein cholesterol; TG, triglycerides; HDL, high density of lipoprotein cholesterol; MCP1, monocyte chemoattractant protein-1; IL6, interleukin 6; TNFα, tumour necrosis factor α.

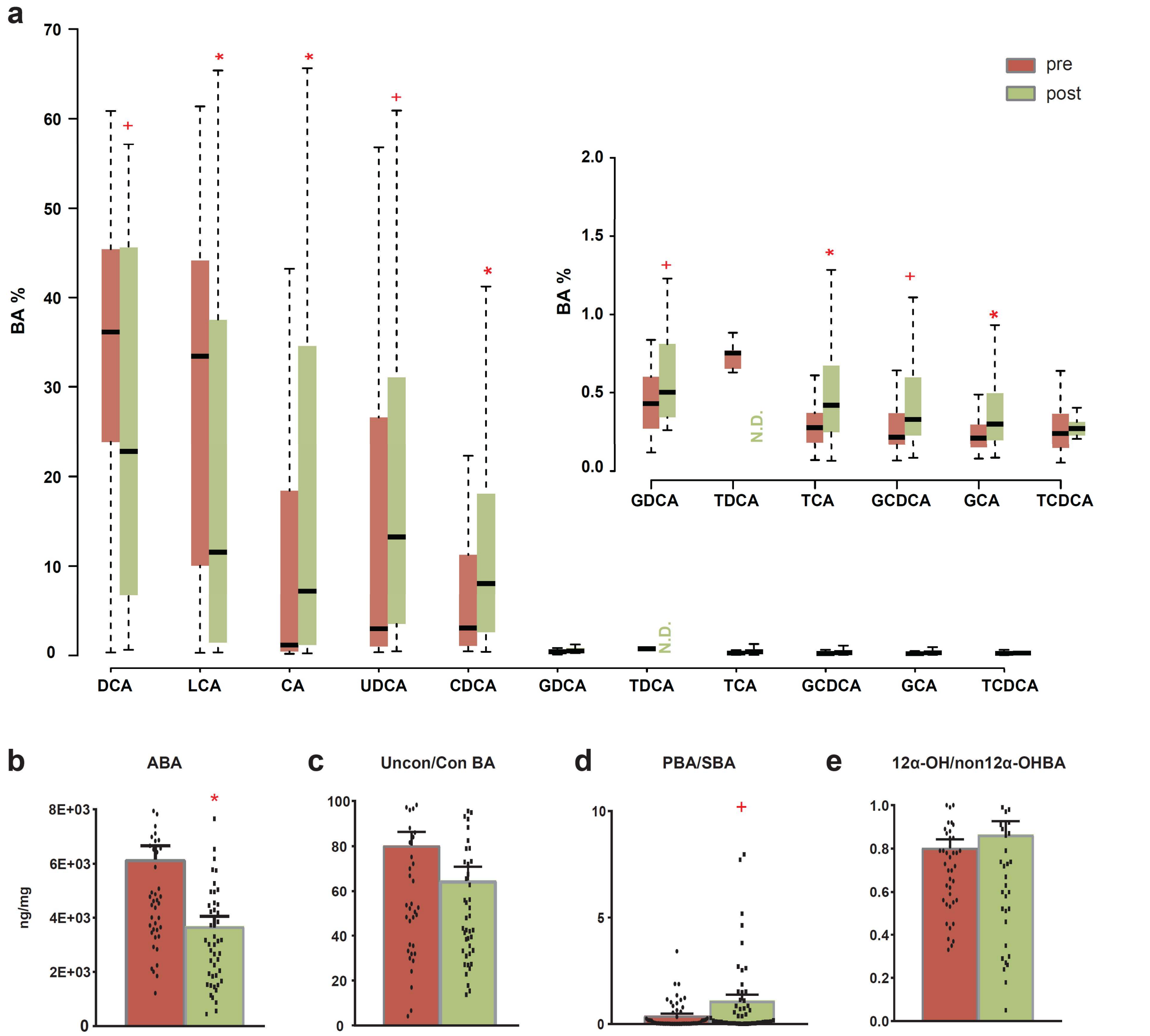

Supplementary Figure 4. Faecal BA levels pre- and post-treatment in the Acarbose arm. (a) Percentage of different BA species in faecal samples. Plotted boxes are interquartile ranges. Dark lines in the boxes indicate medians, the lowest and highest values within 1.5 times IQR from the first and third quartiles. (b) Total amount of all detected faecal BAs. (c) UnconBA/ConBA ratio of faecal BAs. (d) PBA/SBA ratio of faecal BAs. (e) 12α-OH /non 12α-OHBA of faecal BAs. paired Wilcoxon rank-sum test, * P<0.01, + P<0.05, n=50; bar plot is shown as mean ± S.D.; N.D., not detected.

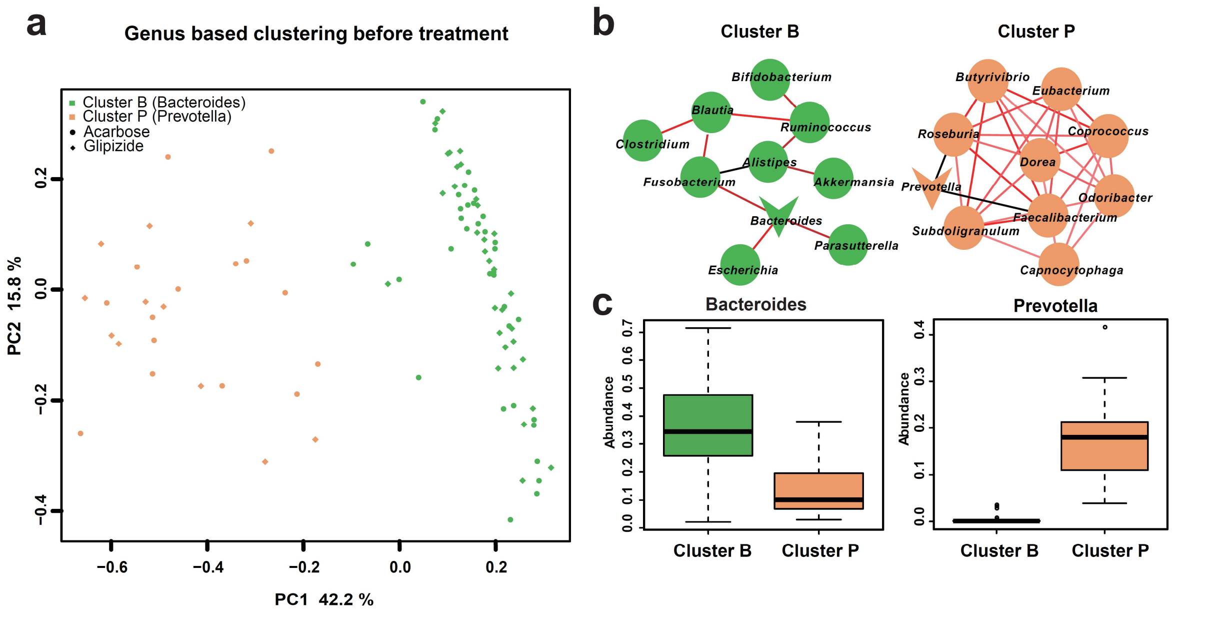

Supplementary Figure 5. Enterotype-like clustering of the gut microbiota in treatment-naïve T2D patients. (a) PCA based on genera in all samples before treatment in both arms. (**b**) Predominant genera in the two microbiome clusters. Arrow heads indicate the representative driver genus in each cluster. Lines between genera indicate the edges between taxa. Red lines indicate positive correlations, and black lines indicate negative correlations; Spearman correlation, P<0.05. (**c**) Plotted boxes show the abundances of driver genera in each cluster, *Bacteroides* or *Prevotella,* Cluster B, n=68; Cluster P, n=26.

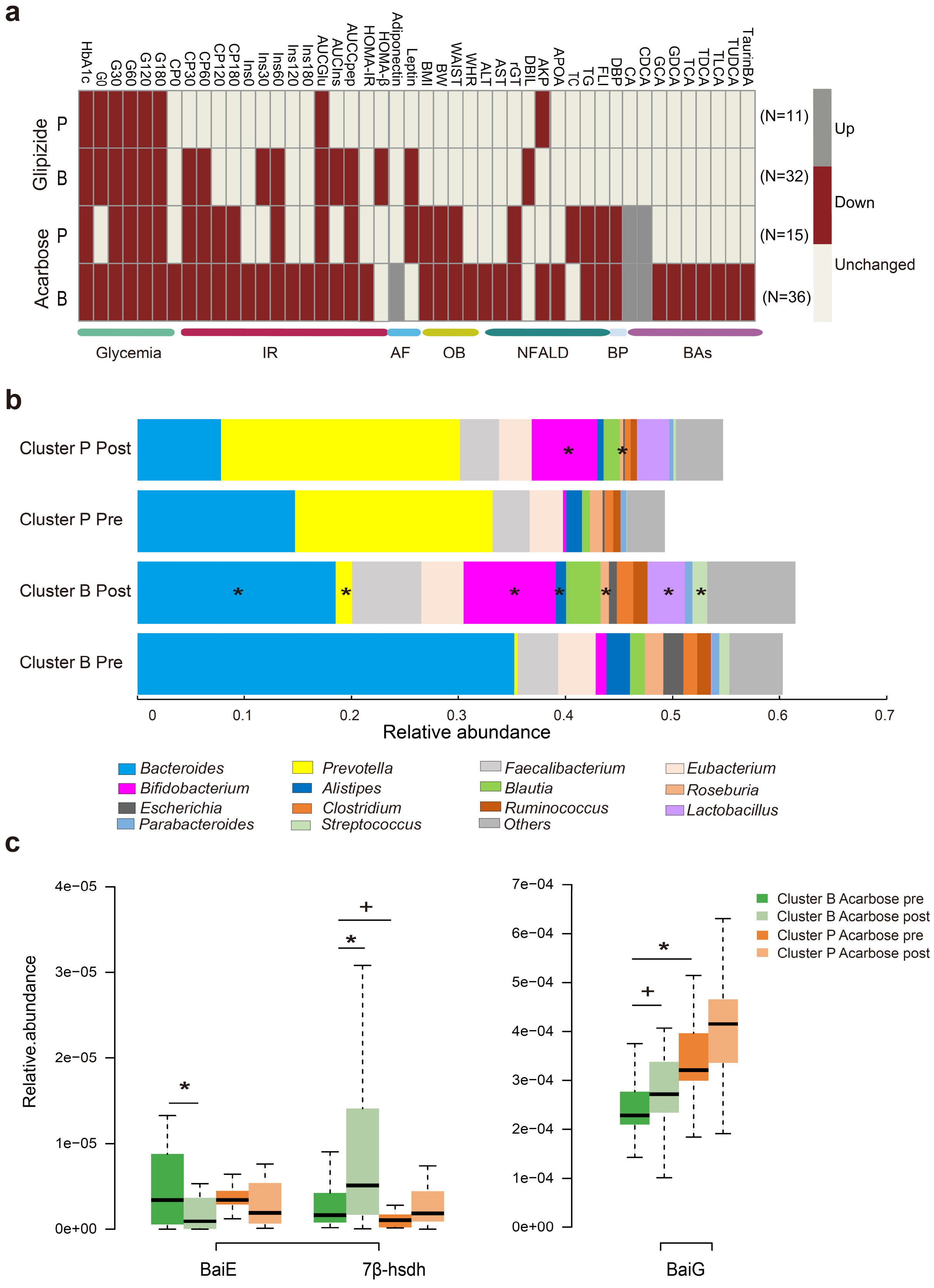
a
HbA1c
G0
G30
G60
G120
G180
CP0
CP30
CP60
CP120
CP180
Ins0
Ins30
Ins60
Ins120
Ins180
AUCGlu
AUCIns
AUCCpep
HOMA-IR
HOMA-β
Adiponectin
Leptin
BMI
BW
WAIST
WHR
ALT
AST
rGT
DBIL
AKP
APOA
TC
TG
FLI
DBP
CA
CDCA
GCA
GDCA
TCA
TDCA
TLCA
TUDCA
TaurinBA
Glipizide
Acarbose
P
B
(N=11)
(N=32)
(N=15)
(N=36)
Up
Down
Unchanged
Glycemia
IR
AF
OB
NFALD
BP
BAs
b
Cluster P Post
Cluster P Pre
Cluster B Post
Cluster B Pre
0
0.1
0.2
0.3
0.4
0.5
0.6
0.7
Relative abundance
Bacteroides
Prevotella
Faecalibacterium
Eubacterium
Bifidobacterium
Alistipes
Blautia
Roseburia
Escherichia
Clostridium
Ruminococcus
Lactobacillus
Parabacteroides
Streptococcus
Others
c
Relative.abundance
4e−05
3e−05
2e−05
1e−05
0e+00
7e−04
6e−04
5e−04
4e−04
3e−04
2e−04
1e−04
0e+00
BaiE
7β-hsdh
BaiG
Cluster B Acarbose pre
Cluster B Acarbose post
Cluster P Acarbose pre
Cluster P Acarbose post

Supplementary Figure 6. Plasma BA composition and therapeutic responses to Acarbose treatment differ in patients with different baseline microbiome compositions.

(**a**) Differential effects of Glipizide and Acarbose on metabolic parameters and plasma BA compositions in patients stratified according to their baseline intestinal microbiome. Glycaemia, glycaemia related clinical parameters; IR, insulin secretion/resistance related indices; AF, adipose tissue secreted factors; OB, anthropometry parameters to measure body obesity; NAFLD, parameters to evaluate non-alcohol fatty liver disease; BP, blood pressures; BAs, bile acids; P, Cluster P; B, Cluster B **(b)** Comparison of the genus composition of the gut microbiota in patients belonging to the *Bacteroides* or *Prevotella* driven enterotype-like clusters, pre- and post-treatment with Acarbose. The 15 most abundant genera were plotted, *, genus significantly changed in relative abundances after treatment, $q<0.01$. (**c**) Relative abundances of genes involved in BA metabolism in samples pre- and post- treatment with Acarbose from patients with gut microbiomes belonging to the *Bacteroides* (Cluster B) or *Prevotella* (Cluster P) driven enterotype-like clusters. Plotted boxes are interquartile ranges. Dark lines in the boxes indicate medians, the lowest and highest values within 1.5 times IQR from the first and third quartiles. Cluster B, n=36; Cluster P, n=15, * $P<0.01$, + $P<0.05$.

Supplementary Tables

Supplementary Table 1 Inclusion and exclusion criteria for enrolment of patients

**Inclusion Criteria:**
1. New onset Type 2 Diabetes;7.0mmol/L<FBG<13.0mmol/L, HbA1c≤10%. Without any previous treatment with oral antidiabetic medicines or insulin
2. BMI<=35 kg/m^2
3. Age between 40-65 years old
4. Understand and voluntarily sign an informed consent document prior to any study related assessments/procedures are conducted
**Exclusion Criteria**
1. Having severe digestive diseases that might cause severe constipation, chronic or acute diarrhoea
2. Had gastroenterological or other abdominal surgeries such as cholecystectomy within one year
3. Having severe hepatic and renal failure (ALT>=2.5 fold higher than the normal range, Scr>=132μmol/L), Psychiatric disorders, severe infectious diseases, severe anaemia, severe neutropenia
4. Severe cardiac diseases, New York Heart Association (NYHA) cardiac function evaluation > Ⅲ degree
5. Had acute diabetic ketonuria acidosis and hyperosmotic coma within 3 months
6. Pregnancy
7. Have known hypersensitivity to test medicine (glucosidase inhibitors or sulfonylureas) or any of the inactive ingredients or have frequent/severe allergic reactions to multiple medications
8. Have been enrolled in other clinical trials within 3 months
9. Yogurt ingestion within 1 week and antibiotics usage within 3 months before the first visit for stool sample collection

FBG, fasting blood glucose; **HbA1c**, Haemoglobin A1c; **ALT**, alanine aminotransferase; **Scr**, Serum creatinine

Supplementary Table 2 Clinical indices pre- and post-treatment in both treatment arms

Clinical Index	Unit	Acarbose					Glipizide					Acarbose vs. Glipizide (Wilcoxon test)		
		Pre-treatment		Post-Treatment		P value (PW test)	Pre-treatment		Post-Treatment		P value (PW test)	A vs. G (Pre)	A vs. G (Post)	A vs G (Δ)
		Value (Mean ± SD)	P value (SW test)	Value (Mean ± SD)	P value (SW test)		Value (Mean ± SD)	P value (SW test)	Value (Mean ± SD)	P value (SW test)		P value	P value	P value
Age	years	53±7				NA	54±7				NA	0.40		NA
Sex (male/Total)		34/51				NA	24/43				NA	0.39		NA
BMI	kg/m^2	26.3 ± 3.2	1.26E-01	25.4 ± 3.2	4.08E-03	3.48E-07	26 ± 3.4	7.73E-05	25.7 ± 3.3	2.09E-05	1.13E-01	2.65E-01	9.46E-01	4.21E-03
BW	kg	74.7 ± 10.3	4.23E-01	72.1 ± 10.1	3.64E-01	2.36E-07	71.5 ± 10.7	2.00E-01	70.6 ± 10.2	1.77E-01	1.38E-01	6.72E-02	1.90E-01	2.50E-03
DBP	mmHg	81.6 ± 8.4	1.79E-02	75.2 ± 8.1	3.28E-01	2.99E-05	81 ± 10.2	2.39E-01	76.1 ± 9.1	6.14E-01	1.28E-02	6.75E-01	7.03E-01	4.33E-01
SBP	mmHg	127.2 ± 15.8	1.40E-02	120.5 ± 13	1.18E-01	2.16E-02	131 ± 18.7	2.29E-02	125.4 ± 14.5	2.39E-01	4.36E-02	4.31E-01	1.28E-01	9.97E-01
Waist circumference	cm	91.5 ± 8.5	5.93E-01	88.6 ± 7.3	8.61E-01	3.85E-04	90.7 ± 8.4	8.21E-02	89.5 ± 8.4	1.89E-02	1.44E-01	5.27E-01	8.70E-01	1.51E-01
WHR		0.9 ± 0.1	2.19E-02	0.9 ± 0	5.43E-01	5.31E-03	0.9 ± 0.1	2.37E-01	0.9 ± 0.1	3.60E-01	1.89E-02	4.85E-01	7.80E-01	7.45E-01
HbA1c	%	7.5 ± 0.8	9.48E-03	6.4 ± 0.5	1.79E-01	1.45E-09	7.7 ± 0.9	4.57E-03	6.3 ± 0.6	9.23E-01	1.82E-08	5.41E-01	5.08E-01	4.68E-01
Blood Glucose level 0'	mmol/L	7.6 ± 1.4	1.45E-03	6.6 ± 0.9	1.84E-02	3.58E-07	7.8 ± 1.4	3.99E-02	6.7 ± 1.2	2.44E-03	2.48E-06	2.91E-01	6.65E-01	3.51E-01
Blood Glucose level 30'	mmol/L	10.4 ± 2	5.96E-03	7.6 ± 1.4	2.80E-02	1.43E-09	11.1 ± 1.8	4.95E-04	8.9 ± 2.2	1.31E-01	2.88E-06	5.25E-02	1.62E-03	1.73E-01
Blood Glucose level 60'	mmol/L	13.7 ± 2.2	2.69E-03	9.1 ± 1.8	1.98E-02	8.28E-10	14.6 ± 2.3	5.96E-04	11.5 ± 2.4	5.61E-02	2.60E-07	9.35E-02	1.38E-06	2.90E-03
Blood Glucose level 120'	mmol/L	14.2 ± 2.7	3.35E-04	9.2 ± 1.8	3.19E-04	5.30E-10	14.9 ± 2.8	2.53E-03	10.8 ± 2.8	4.06E-03	6.29E-10	1.64E-01	2.01E-03	1.54E-01
Blood Glucose level 180'	mmol/L	11.4 ± 3	9.05E-06	7.9 ± 2.1	9.56E-04	7.37E-09	12.6 ± 3.2	5.49E-03	8.9 ± 3	5.16E-03	9.80E-10	8.84E-02	2.56E-01	4.74E-01
AUC blood Glucose	mmol/L*min	2241 ± 363	8.16E-03	1507 ± 263	1.47E-02	5.30E-10	2381 ± 351	5.42E-03	1804 ± 359	7.35E-02	6.98E-11	7.70E-02	6.55E-05	4.89E-02
C Peptide 0'	ng/mL	1.2 ± 0.5	5.84E-02	1.1 ± 0.5	1.39E-04	1.12E-02	1.1 ± 0.4	3.24E-02	1.1 ± 0.4	7.22E-01	3.47E-01	4.34E-01	7.36E-01	2.08E-01
C Peptide 30'	ng/mL	1.7 ± 0.8	1.81E-03	1.4 ± 0.6	2.22E-01	1.02E-06	1.6 ± 0.5	8.62E-02	2.1 ± 0.7	1.21E-01	1.38E-06	5.92E-01	1.47E-07	4.91E-12
C Peptide 60'	ng/mL	2.7 ± 1.4	1.52E-02	1.9 ± 0.9	2.55E-02	2.30E-06	2.4 ± 0.9	1.95E-02	2.9 ± 1	1.19E-02	3.42E-04	3.63E-01	1.28E-08	4.99E-09
C Peptide 120'	ng/mL	3.9 ± 1.7	4.57E-03	2.8 ± 1.1	4.83E-01	8.13E-08	3.8 ± 1.3	7.39E-01	4.2 ± 1.4	5.72E-01	2.14E-02	9.44E-01	3.54E-08	9.39E-08
C Peptide 180'	ng/mL	3.6 ± 1.6	7.96E-05	2.5 ± 1.2	4.81E-03	7.49E-08	3.5 ± 1.2	8.69E-01	3.8 ± 1.2	4.36E-01	1.04E-01	4.81E-01	7.73E-08	1.03E-06
AUC C Peptide	ng/mL*min	528 ± 210	1.96E-02	384 ± 143	8.05E-01	1.94E-08	501 ± 154	2.57E-01	575 ± 161	1.54E-01	1.95E-03	9.78E-01	1.94E-07	4.78E-10
Insulin 0'	pmol/L	83.8 ± 45.1	7.16E-05	68.6 ± 44.2	6.44E-05	2.12E-02	80.7 ± 47	3.62E-03	76.3 ± 48.6	2.20E-03	6.03E-01	5.92E-01	7.76E-01	1.57E-01
Insulin30'	pmol/L	138.9 ± 79.8	1.67E-05	100.5 ± 58	1.69E-04	9.12E-04	135.2 ± 65.7	1.15E-02	194.1 ± 112.6	1.26E-05	2.61E-05	8.93E-01	2.26E-06	1.29E-07
Insulin 60'	pmol/L	258.5 ± 163.2	2.63E-07	157.4 ± 97.5	1.26E-03	2.73E-05	226.6 ± 121.2	2.35E-01	283.3 ± 172.5	1.48E-03	6.03E-03	3.93E-01	1.26E-06	1.03E-06
Insulin120'	pmol/L	361.8 ± 224.1	3.39E-07	210.6 ± 119.1	2.11E-07	3.85E-06	318.8 ± 156.3	2.19E-03	378.9 ± 223.6	3.84E-02	5.72E-02	6.39E-01	2.53E-06	7.78E-06
Insulin180'	pmol/L	286.7 ± 180.6	2.22E-04	169.2 ± 127	5.77E-03	1.63E-06	277.8 ± 151.6	5.86E-02	309.6 ± 170.3	1.72E-04	1.31E-01	7.66E-01	6.78E-06	1.30E-05
AUC Insulin*	pmol/L*min	1029.9 ± 575.2	1.50E-06	637.1 ± 342.6	6.61E-04	2.41E-06	940.2 ± 431.7	1.62E-01	1139.6 ± 608.9	3.83E-04	1.90E-02	6.97E-01	1.62E-06	9.54E-08
LDL	mmol/L	1.2 ± 0.4	3.78E-08	1.2 ± 0.3	5.71E-05	2.76E-01	1.2 ± 0.4	1.15E-08	1.3 ± 0.6	9.43E-09	5.35E-01	5.00E-01	8.64E-01	7.68E-01
HDL	mmol/L	3.1 ± 1	6.54E-03	3 ± 1	5.66E-01	4.84E-01	3.1 ± 0.8	8.73E-01	2.9 ± 0.8	1.93E-01	3.58E-02	4.92E-01	5.14E-01	2.07E-01
APOA	g/L	1.3 ± 0.2	7.22E-01	1.2 ± 0.2	6.96E-01	2.46E-03	1.4 ± 0.1	5.74E-01	1.3 ± 0.2	9.22E-01	2.25E-01	7.97E-01	2.35E-01	1.01E-01
APOB	g/L	1.1 ± 0.3	3.02E-01	1 ± 0.3	8.29E-01	2.98E-01	1 ± 0.2	4.69E-01	1 ± 0.2	8.76E-01	2.36E-01	7.87E-01	9.15E-01	8.76E-01
TC	mmol/L	5.1 ± 1.2	3.79E-02	4.8 ± 1.2	6.43E-02	7.47E-03	4.9 ± 1	3.73E-02	4.8 ± 0.8	7.28E-01	2.28E-01	6.96E-01	7.12E-01	3.18E-01

TG	mmol/L	2.5 ± 2.1	6.55E-09	1.6 ± 1.2	2.11E-09	2.39E-05	2 ± 1.2	2.17E-05	1.7 ± 0.8	1.27E-03	3.82E-01	4.59E-01	9.27E-02	3.19E-03
AST	IU/L	30.6 ± 24.9	2.60E-11	24.4 ± 15.3	6.32E-10	3.96E-03	25.9 ± 14.5	1.02E-07	20.8 ± 5.3	3.74E-06	4.36E-02	1.73E-01	5.96E-01	4.33E-01
AKP	IU/L	73.6 ± 23.3	1.94E-03	64.5 ± 22.2	3.85E-04	1.26E-04	69.6 ± 14.8	9.12E-01	66.1 ± 16.8	3.98E-01	2.74E-02	8.99E-01	2.92E-01	3.26E-01
ALB	g/L	48.5 ± 31.4	1.74E-14	42.9 ± 3.1	2.57E-01	1.61E-02	43.5 ± 3	2.81E-01	43.4 ± 3.3	1.13E-02	6.11E-01	5.07E-01	9.87E-01	1.32E-01
ALT	IU/L	38.6 ± 27.3	2.19E-07	33.7 ± 37.1	1.14E-11	1.38E-02	33 ± 25.5	4.82E-08	24.1 ± 11.1	2.29E-08	2.25E-02	1.20E-01	2.96E-01	6.16E-01
γGT	IU/L	48 ± 53.9	2.22E-10	30.1 ± 33.5	6.42E-11	3.03E-06	33.5 ± 20.4	6.42E-05	27.9 ± 22.8	4.54E-08	1.70E-02	2.96E-01	6.27E-01	2.38E-02
Uric acid	umol/L	5 ± 1	8.42E-01	4.8 ± 1.4	3.19E-01	1.83E-01	5.2 ± 1.3	5.35E-01	5.2 ± 1.5	2.02E-01	9.60E-01	4.31E-01	3.26E-01	2.86E-01
Creatine	umol/L	67.4 ± 14.3	8.60E-01	67.6 ± 14.6	2.02E-01	5.21E-01	67.2 ± 14	6.74E-01	66.2 ± 13.1	2.47E-01	6.84E-01	8.82E-01	7.17E-01	4.80E-01
Bun	mmol/L	308.3 ± 73.3	2.47E-01	328.5 ± 60.2	1.98E-01	6.85E-03	292.5 ± 68.5	1.96E-02	294.5 ± 50.7	5.02E-01	4.20E-01	1.77E-01	1.10E-02	1.49E-01
HOMA-IR*	uIU*mmol	3.7 ± 2.3	2.71E-05	2.6 ± 1.8	4.16E-05	2.25E-03	3.6 ± 2.1	8.33E-03	2.9 ± 2	5.24E-04	5.05E-02	8.68E-01	6.38E-01	2.39E-01
HOMA-β*	%	55 ± 32.9	1.65E-07	60 ± 43.8	1.07E-06	9.88E-01	52.5 ± 37.6	1.06E-05	70.9 ± 61.9	1.19E-07	7.38E-03	1.94E-01	8.44E-01	4.07E-02
FLI		334 ± 555	6.61E-11	101 ± 147	5.46E-11	1.92E-09	237 ± 341	8.97E-09	162 ± 271	4.70E-10	1.33E-02	3.52E-01	4.00E-01	5.85E-13
ΔIns30/G30*	uIU/mmol	2.2 ± 3.5	7.69E-06	31 ± 111	2.13E-13	1.19E-02	2 ± 1.8	1.80E-01	10.9 ± 15.6	1.09E-09	1.64E-08	6.80E-01	1.53E-01	2.75E-02
CRP	ug/mL	12.6 ± 15.4	2.10E-11	26.8 ± 64.2	6.61E-13	6.29E-01	16.8 ± 40.1	1.15E-12	12.4 ± 12.6	3.52E-07	3.17E-01	2.85E-01	9.63E-01	7.79E-01
TNF α	pg/mL	4.5 ± 2.1	5.39E-05	4.7 ± 2.3	1.81E-02	4.23E-01	5.3 ± 3.8	4.31E-07	4.7 ± 2.5	1.45E-04	1.05E-01	2.30E-01	9.78E-01	6.59E-02
LBP (LPS binding protein)	ng/mL	18.4 ± 5.8	2.83E-01	17.4 ± 6.4	1.37E-01	7.33E-01	22.1 ± 8.7	8.24E-02	18.8 ± 6.2	5.88E-02	3.47E-02	2.87E-02	6.90E-01	1.68E-01
IL6	pg/mL	2.4 ± 4.5	1.43E-11	4.3 ± 8.4	1.35E-10	7.61E-02	3.5 ± 5.2	2.60E-09	1.8 ± 1.9	4.26E-08	1.68E-03	2.95E-02	1.04E-01	2.51E-04
IL8	ng/mL	13.6 ± 19.2	1.64E-10	13 ± 20.1	1.34E-10	9.88E-01	22.2 ± 31.4	1.18E-08	11.6 ± 17.3	1.33E-09	3.47E-02	1.47E-01	4.55E-01	7.52E-02
mpo	ng/mL	380 ± 351	5.18E-06	334 ± 302	6.98E-07	6.57E-01	441 ± 360	3.53E-05	342 ± 373	1.46E-08	2.48E-02	2.05E-01	8.95E-01	1.69E-01
MCP1	pg/mL	235.6 ± 89.3	7.34E-01	255.5 ± 90.2	9.36E-01	2.79E-03	256 ± 154	5.39E-09	263 ± 156	2.62E-09	3.59E-01	8.38E-01	4.55E-01	1.46E-01
PAI1	pg/mL	55585 ± 18211	9.32E-02	55291 ± 22546	7.20E-05	7.37E-01	61351 ± 23327	1.05E-03	54972 ± 21238	1.87E-03	5.20E-02	4.59E-01	8.88E-01	1.47E-01
Endothelin-1	pg/mL	12.8 ± 3	1.15E-05	13.7 ± 2.8	6.15E-03	1.48E-02	12.9 ± 2.8	1.40E-03	13.3 ± 3.5	1.08E-06	2.84E-01	8.46E-01	3.56E-01	3.19E-01
FetuinA	ug/mL	338.8 ± 50.7	5.94E-01	406 ± 109	4.18E-08	2.76E-07	327.2 ± 43.1	5.02E-01	392.2 ± 84.7	2.79E-07	1.73E-10	2.20E-01	6.81E-01	3.95E-01
FGF19	MFI	22.5 ± 4.1	7.17E-01	19.6 ± 4.2	1.70E-05	1.34E-05	23.8 ± 3.9	2.34E-01	21.4 ± 4.8	2.21E-03	2.26E-03	1.68E-01	3.12E-01	9.75E-01
FGF21	MFI	141.1 ± 68.8	2.87E-08	99.4 ± 59	4.77E-09	3.08E-06	146 ± 107	1.43E-04	128.2 ± 75.3	5.46E-05	1.63E-01	7.41E-01	6.97E-01	1.38E-02
FGF23	MFI	44.2 ± 6.7	3.70E-02	39 ± 9.6	2.74E-04	1.73E-03	45.5 ± 10.8	4.16E-05	43.1 ± 14.1	9.45E-08	8.93E-03	8.37E-01	5.23E-01	4.57E-01
Adiponectin	pg/mL	5296311 ± 3989030	3.94E-06	6659153 ± 6643764	1.36E-07	3.32E-03	5812437 ± 2657385	1.48E-05	5866403 ± 2998557	1.03E-01	5.38E-01	5.39E-02	6.85E-01	1.40E-01
Leptin	pg/mL	6566 ± 6138	9.52E-07	5881 ± 8247	9.33E-11	1.03E-02	6408 ± 6743	2.32E-07	6485 ± 6630	6.66E-08	3.39E-01	6.52E-01	1.90E-01	1.88E-02

Data are presented as mean ± SD.

P value (SW test), P values calculated by Shapiro–Wilk test; P value (PW test), P values calculated by paired Wilcoxon rank-sum test; *, the unit of insulin is converted to uIU/L. MFI, mean fluorescence intensity determined by using the Luminex 200™ instrument.

A vs G (Pre), comparison of pre-treatment values between Acarbose and Glipizide arms; A vs G (Post), comparison of post treatment values between Acarbose and Glipizide arms.; A vs G（Δ), comparison of changes induced by each treatment arm

BW, body weight; **DBP**, diastolic blood pressure; **SBP**, systolic blood pressure; **WHR**, waist-to-hip ratio; **HbA1c**, Haemoglobin A1c;

AUC Insulin, area under curve value of plasma insulin level during meal test; **AUC C peptide**, area under curve value of plasma C peptide level during meal test; **AUC Glucose**, area under curve value of blood glucose level during meal test;

LDL, low-density lipoprotein; **HDL**, high-density lipoprotein; **APOA**, apolipoprotein A; **APOB,** apolipoprotein B; **TC**, total cholesterol; **TG**, triglyceride; **AST**, aspartate aminotransferase; **ALB**, albumin; **ALT**, alanine transaminase; **γGT,** gamma-glutamyltransferase; **Bun**, blood urea nitrogen; **HOMA-IR**, the homeostasis model assessment-insulin resistance; **HOMA-β,** the homeostasis model assessment-β-cell function; **FLI**, fatty liver index; **ΔIns30/G30**= (Insulin 30'-Insulin 0')/(blood Glucose level 30'-blood Glucose level 0') during meal test. **CRP**, C-reactive protein; **TNFα,** tumour necrosis factor α; **LBP,** lipopolysaccharides binding protein; **IL**, interleukin; **MCP-1**, monocyte chemoattractant protein-1; **PAI1**, plasminogen activator inhibitor-1; FGF, fibroblast growth factor.

Supplementary Table 3 Plasma bile acid pre-and post-treatment in both treatment arms

Plasma bile acid (nmol/L)	Acarbose				Glipizide				Acarbose pre vs. Glipizide pre
	**Pre-Treatment**	**Post-Treatment**	***P* value (Absolute value)**	***P* value (% of ABA)**	**Pre-Treatment**	**Post-Treatment**	***P* value (Absolute value)**	***P* value (% of ABA)**	***P* value (Absolute value)**
**Primary BAs**									
CA	100±133	211±258	2.00E-03	0.002	134±268	119±200	5.69E-01	7.29E-01	5.50E-01
CDCA	297±328	680±550	3.20E-05	4.20E-08	290±396	396±870	8.24E-01	2.23E-01	5.93E-01
GCA	366±629	249±432	4.00E-03	9.01E-05	401±781	329±617	1.87E-01	2.18E-01	6.24E-01
TCA	40±86	22±45	1.00E-03	2.56E-04	34±76	32±71	9.80E-01	2.48E-01	2.48E-01
TCDCA	119±187	76±77	1.59E-01	8.59E-04	92±145	89±140	6.75E-01	8.24E-01	5.06E-01
GCDCA	1124±1263	959±992	2.87E-01	4.20E-03	1156±1568	1001±1341	3.33E-01	2.18E-02	9.02E-01
**Secondary BAs**									
UDCA	179±282	256±346	1.29E-01	0.407	234±336	245±395	2.38E-01	8.14E-01	9.53E-01
TUDCA	16±27	8±8	3.30E-02	2.94E-04	14±25	9±13	2.96E-01	5.60E-01	4.57E-01
GUDCA	112±135	87±93	2.87E-01	0.061	126±175	96±110	2.63E-01	3.78E-01	8.71E-01
DCA	184±191	142±166	2.30E-01	0.039	198±219	183±160	7.22E-01	9.61E-01	9.46E-01
LCA	18±20	13±13	5.50E-02	0.234	15±12	16±11	7.10E-01	4.13E-01	9.84E-01
TDCA	38±75	13±15	7.91E-04	3.07E-04	29±52	31±39	6.82E-01	1.28E-01	8.21E-01
GDCA	468±806	175±292	2.00E-03	5.17E-05	470±907	421±575	9.95E-01	4.08E-01	7.65E-01
GLCA	8±13	5±5	3.01E-01	0.448	12±27	10±15	9.02E-01	5.44E-01	2.36E-01
TLCA	8±17	4±4	7.80E-02	0.080	7±11	7±7	6.56E-01	8.24E-01	5.99E-01
**BA index**									

UnconBA/ConBA	0.7±0.6	1.4±1.3	9.89E-06		0.5±0.4	0.8±1.1	1.02E-01		6.77E-01
12α-OH/non 12α-OHBA	0.7±0.5	0.5±0.3	2.00E-03		0.7±0.4	0.8±0.5	5.36E-01		5.82E-01
PBA/SBA	2.2±1.5	4.9±4.8	3.57E-05		1.9±1.2	1.8±1.5	3.46E-01		3.12E-01
TaurineBA	222±371	121±126	1.35E-02		178±286	168±244	7.86E-01		5.66E-01
ABA	3342±3265	3111±2135	7.88E-01		3461±4011	3219±3226	8.24E-01		9.09E-01

Data are presented as mean ± SD; 91 of 94 have plasma BA profiles assayed. Unit of all BAs is nmol/L.

CA, cholic acid; GCA, glycocholic acid; TCA, taurocholic acid

CDCA, chenodeoxycholic acid; TCDCA, taurochenodeoxycholic acid; GCDCA, glycochenodeoxycholic acid

UDCA, ursodeoxycholic acid; TUDCA, tauroursodeoxycholic acid; GUDCA, glycoursodeoxycholic acid

DCA, deoxycholic acid; GDCA, glycodeoxycholic acid; TDCA, taurodeoxycholic acid

LCA, lithocholic acid, GLCA; glycolithocholic acid, TLCA; taurolithocholic acid

UnconBA, unconjugated bile acids; ConBA, conjugated bile acids;

12α-OH, 12α-hydroxylated; PBA, primary bile acids; SBA, secondary bile acids; ABA, sum of all bile acids

1. 12 α-OH/non12α-OHBA = GCA+CA+TCA+GDCA+DCA+TDCA) / (TUDCA+CDCA+TCDCA+GLCA+LCA+UDCA+GCDCA+TLCA+GUDCA

2. UnconBA/ConBA = CA + UDCA + CDCA + DCA + LCA) / (GCDCA + GDCA + GUDCA + GCA + TUDCA + TCA + TCDCA + TDCA + GLCA + TLCA

3. PBA/SBA = GCDCA+ TCDCA + CDCA + CA + TCA + GCA) / (UDCA + GUDCA + TUDCA + DCA + TDCA + GDCA + LCA + GLCA + TLCA

4. TaurineBA = TUDCA + TCA + TCDCA + TDCA + TLCA

5. ABA=UDCA + GCDCA + GDCA + GUDCA + CA + GCA + TUDCA + TCA + CDCA + DCA + TCDCA + TDCA + GLCA + LCA + TLCA

P value (Absolute value) indicates the *P* values were calculated based on absolute values of plasma BAs.

P value (% of ABA) indicates the *P* values were calculated based on percentage of each BA species in ABA.

Supplementary Table 4 Generalized estimated equations analysis for the contribution of plasma bile acid composition to clinical outcomes

Clinical index	12α-OH/non 12α-OHBA			PBA/SBA			UnconBA/ConBA		
	β	***P* value**	***q* value**	***β***	***P* value**	***q* value**	***β***	***P* value**	***q* value**
HOMA-IR	0.012	9.14E-01	9.26E-01	-0.104	2.18E-01	3.65E-01	-0.347	7.72E-07	1.85E-05
AUCIns	0.096	2.89E-01	4.96E-01	-0.213	4.88E-03	7.70E-02	-0.316	1.09E-05	1.31E-04
AUCGlu	0.057	5.61E-01	7.91E-01	-0.151	5.17E-02	1.77E-01	-0.314	1.41E-04	1.13E-03
ΔIns30/G30	-0.122	1.07E-01	2.85E-01	0.193	2.28E-01	3.65E-01	-0.019	7.56E-01	9.08E-01
HOMA-β	0.010	9.26E-01	9.26E-01	-0.030	7.76E-01	8.86E-01	-0.231	2.49E-03	8.54E-03
AUCCpep	0.137	9.08E-02	2.85E-01	-0.192	1.81E-02	9.44E-02	-0.298	8.53E-04	4.10E-03
HbA1c	-0.032	6.93E-01	8.69E-01	-0.113	1.87E-01	3.65E-01	-0.205	1.38E-02	3.67E-02
WHR	0.144	5.51E-02	2.65E-01	-0.012	8.32E-01	9.08E-01	-0.006	9.31E-01	9.31E-01
BW	0.080	5.27E-03	4.22E-02	-0.073	1.72E-02	9.44E-02	-0.094	4.72E-04	2.83E-03
SBP	-0.040	7.24E-01	8.69E-01	0.099	4.05E-01	4.86E-01	-0.161	2.57E-02	5.61E-02
DBP	0.072	5.23E-01	7.84E-01	-0.103	2.10E-01	3.65E-01	-0.186	4.06E-02	7.50E-02
FLI	-0.068	2.66E-01	4.94E-01	-0.092	2.09E-01	3.65E-01	-0.169	1.15E-02	3.46E-02
TG	-0.074	1.56E-01	3.74E-01	-0.329	6.41E-03	7.70E-02	-0.219	1.97E-03	7.89E-03
TC	0.112	1.97E-01	4.31E-01	-0.158	2.69E-02	1.08E-01	-0.080	3.28E-01	5.02E-01
LDL	-0.005	9.25E-01	9.26E-01	-0.038	4.04E-01	4.86E-01	-0.065	4.65E-01	6.53E-01
HDL	0.137	9.74E-02	2.85E-01	-0.002	9.76E-01	9.76E-01	-0.049	4.90E-01	6.53E-01
FGF19	0.254	3.84E-03	4.22E-02	-0.147	1.97E-02	9.44E-02	-0.173	3.80E-02	7.50E-02
FGF21	-0.020	7.83E-01	8.95E-01	0.039	1.55E-01	3.65E-01	0.054	3.17E-01	5.02E-01
LBP	-0.085	2.68E-01	4.94E-01	-0.120	2.20E-01	3.65E-01	-0.009	9.17E-01	9.31E-01
TNFa	-0.154	1.24E-02	7.45E-02	0.087	3.86E-01	4.86E-01	0.053	6.60E-01	8.34E-01
MCP1	-0.243	1.49E-08	3.58E-07	0.005	9.23E-01	9.63E-01	0.124	2.56E-02	5.61E-02
IL6	-0.171	8.43E-02	2.85E-01	0.056	3.62E-01	4.86E-01	0.023	8.64E-01	9.31E-01
Adiponectin	0.042	6.41E-01	8.55E-01	-0.044	3.81E-01	4.86E-01	-0.011	8.38E-01	9.31E-01
Leptin	0.042	4.48E-01	7.17E-01	0.131	1.88E-01	3.65E-01	0.054	3.35E-01	5.02E-01

β, correlation coefficients in generalized estimating equations model.

Supplementary Table 5 Metagenomic data production from both treatment arms

	Mean Clean reads	Mean Mapping rate (%)	Abundance changed after treatment		Abundance changed after treatment	
			Gene number (P<0.01)	mOTU number (P<0.05)	Gene number (q<0.01)	mOTU number (q<0.01)
**Acarbose**	7.7Gb	80.07	472847	162	141382	69
**Glipizide**	7.4Gb	81.12	6711	5	0	0

Q value indicates an FDR adjusted p-value by Benjamini & Hochberg (BH) method.

Supplementary Table 6 List of mOTUs that changed in relative abundance in response to treatment in the Acarbose and the Glipizide arms ($P<0.05$)

**Acarbose**				
**mOTU ID**	***P* value**	***q* value**	**Relative Abundance**	
			**Pre**	**Post**
Prevotella copri	8.17E-03	2.67E-02	1.03E-01	1.34E-01
Bacteroides dorei/vulgatus	2.35E-07	1.01E-05	8.30E-02	3.50E-02
Bacteroides plebeius	2.21E-03	9.79E-03	5.45E-02	2.97E-02
Bacteroides uniformis	2.02E-04	1.42E-03	3.34E-02	1.65E-02
Alistipes putredinis	2.35E-07	1.01E-05	2.25E-02	7.08E-03
Bacteroides stercoris	8.48E-05	6.95E-04	2.25E-02	8.74E-03
motu linkage group 158	3.20E-06	8.03E-05	2.10E-02	3.01E-03
Eubacterium eligens	4.34E-04	2.67E-03	1.92E-02	7.48E-03
motu linkage group 284	7.96E-09	2.40E-06	1.42E-02	1.92E-03
motu linkage group 115	1.18E-02	3.30E-02	1.26E-02	9.85E-03
Parabacteroides distasonis	1.08E-02	3.15E-02	1.22E-02	1.73E-02
Bacteroides thetaiotaomicron	1.73E-05	2.16E-04	1.16E-02	3.19E-03
motu linkage group 104	2.02E-02	4.94E-02	1.13E-02	2.40E-02
motu linkage group 325	1.23E-06	3.69E-05	1.10E-02	5.27E-04
Bacteroides xylanisolvens	1.95E-02	4.81E-02	1.02E-02	6.07E-03
motu linkage group 536	1.27E-02	3.42E-02	9.70E-03	4.09E-03
motu linkage group 260	6.92E-03	2.30E-02	9.40E-03	2.14E-03
Roseburia inulinivorans	1.52E-05	2.07E-04	9.25E-03	3.09E-03
motu linkage group 134	2.33E-03	1.00E-02	9.05E-03	2.19E-03
motu linkage group 290	1.14E-02	3.24E-02	7.95E-03	3.31E-03
motu linkage group 258	8.54E-06	1.71E-04	7.94E-03	2.09E-03
motu linkage group 206	1.44E-07	8.67E-06	6.90E-03	7.53E-04
Streptococcus salivarius	6.38E-06	1.37E-04	6.43E-03	1.32E-02
motu linkage group 490	2.75E-04	1.88E-03	6.39E-03	3.66E-03
motu linkage group 131	9.58E-04	5.06E-03	6.34E-03	3.49E-03
Bifidobacterium catenulatum-Bifidobacterium pseudocatenulatum complex	1.46E-05	2.07E-04	6.24E-03	3.11E-02
motu linkage group 316	4.79E-04	2.77E-03	6.05E-03	1.92E-02
Ruminococcus sp. 5 1 39BFAA	5.29E-04	2.95E-03	5.62E-03	3.18E-03
Roseburia intestinalis	2.24E-05	2.41E-04	5.24E-03	3.45E-04
Alistipes shahii	5.13E-03	1.82E-02	4.78E-03	3.42E-03
Collinsella aerofaciens	7.29E-05	6.45E-04	4.72E-03	1.43E-02
Bifidobacterium longum	5.38E-07	2.02E-05	4.49E-03	5.35E-02
Bacteroides caccae	3.09E-03	1.26E-02	4.06E-03	1.96E-03
motu linkage group 349	2.05E-08	3.09E-06	3.89E-03	9.57E-04
motu linkage group 265	5.52E-03	1.91E-02	3.79E-03	1.31E-03
motu linkage group 471	3.33E-03	1.29E-02	3.62E-03	1.17E-03
motu linkage group 520	2.04E-03	9.29E-03	3.43E-03	1.70E-03
Capnocytophaga sp. oral taxon 329	6.97E-03	2.30E-02	3.41E-03	1.25E-03

Clostridium sp. L2-50	2.04E-04	1.42E-03	3.35E-03	4.08E-05
Bacteroides intestinalis	9.35E-06	1.76E-04	3.31E-03	5.93E-04
motu linkage group 362	4.25E-03	1.62E-02	3.26E-03	6.60E-04
motu linkage group 366	1.21E-04	9.56E-04	3.26E-03	1.41E-03
butyrate-producing bacterium	1.24E-02	3.39E-02	3.20E-03	1.30E-02
Bacteroides finegoldii	8.55E-05	6.95E-04	3.13E-03	5.80E-04
motu linkage group 70	3.92E-04	2.51E-03	3.11E-03	9.45E-03
motu linkage group 254	1.64E-03	7.86E-03	3.08E-03	6.71E-04
[Ruminococcus] torques	1.76E-03	8.18E-03	3.02E-03	1.52E-02
Bifidobacterium adolescentis	7.08E-05	6.45E-04	2.89E-03	4.05E-02
motu linkage group 552	1.06E-02	3.13E-02	2.85E-03	1.65E-03
motu linkage group 551	1.83E-04	1.37E-03	2.84E-03	2.57E-03
Ruminococcus lactaris	1.86E-05	2.18E-04	2.68E-03	9.22E-04
motu linkage group 394	4.57E-04	2.70E-03	2.62E-03	8.65E-04
motu linkage group 456	1.45E-05	2.07E-04	2.31E-03	8.44E-04
Odoribacter splanchnicus	3.73E-06	8.63E-05	2.21E-03	7.71E-04
Bilophila wadsworthia	1.28E-07	8.67E-06	2.21E-03	7.09E-04
Veillonella atypica	1.25E-02	3.39E-02	2.19E-03	1.30E-03
Fusobacterium mortiferum	3.33E-03	1.29E-02	2.17E-03	2.87E-04
motu linkage group 301	1.09E-02	3.15E-02	1.90E-03	1.20E-03
Clostridium bolteae	9.17E-08	8.67E-06	1.87E-03	1.24E-04
Eubacterium ventriosum	1.87E-04	1.37E-03	1.86E-03	5.92E-04
motu linkage group 282	1.60E-02	4.09E-02	1.82E-03	2.62E-04
motu linkage group 225	1.05E-05	1.80E-04	1.78E-03	1.60E-03
motu linkage group 135	1.01E-02	3.04E-02	1.73E-03	1.02E-03
Eubacterium siraeum	2.87E-03	1.18E-02	1.67E-03	2.90E-04
motu linkage group 443	1.50E-04	1.16E-03	1.63E-03	7.56E-04
motu linkage group 238	4.47E-04	2.69E-03	1.59E-03	1.98E-04
motu linkage group 268	1.21E-02	3.34E-02	1.58E-03	9.91E-05
Roseburia hominis	1.65E-05	2.16E-04	1.53E-03	5.05E-04
motu linkage group 306	9.72E-03	2.97E-02	1.51E-03	5.28E-04
Veillonella parvula	5.71E-03	1.95E-02	1.47E-03	8.07E-03
motu linkage group 118	4.99E-04	2.83E-03	1.46E-03	3.86E-04
Alistipes sp. HGB5	1.22E-03	6.00E-03	1.42E-03	7.72E-04
motu linkage group 431	1.46E-05	2.07E-04	1.32E-03	4.96E-04
Bacteroides clarus	4.34E-03	1.63E-02	1.27E-03	1.90E-04
motu linkage group 415	5.39E-03	1.89E-02	1.20E-03	2.64E-04
motu linkage group 507	1.77E-03	8.18E-03	1.06E-03	5.65E-04
motu linkage group 274	1.38E-02	3.65E-02	9.50E-04	8.81E-04
motu linkage group 470	2.51E-05	2.60E-04	8.73E-04	1.14E-04
motu linkage group 646	8.26E-03	2.67E-02	8.50E-04	2.45E-03
motu linkage group 145	9.15E-03	2.87E-02	8.36E-04	1.55E-06
motu linkage group 573	6.54E-04	3.52E-03	7.99E-04	1.24E-02
motu linkage group 624	1.89E-05	2.18E-04	7.69E-04	5.56E-04
unclassified Lachnospiraceae	2.87E-03	1.18E-02	7.64E-04	5.82E-05

motu linkage group 651	1.40E-02	3.68E-02	6.62E-04	3.25E-04
Blautia hansenii	3.17E-03	1.27E-02	6.02E-04	9.63E-05
Megasphaera elsdenii	3.21E-04	2.10E-03	5.49E-04	2.61E-03
motu linkage group 600	3.22E-03	1.27E-02	5.09E-04	5.72E-05
motu linkage group 495	1.09E-03	5.50E-03	4.64E-04	1.47E-04
motu linkage group 497	6.25E-03	2.11E-02	4.61E-04	3.16E-04
motu linkage group 586	2.89E-05	2.90E-04	4.55E-04	4.09E-05
motu linkage group 491	9.09E-03	2.87E-02	4.48E-04	3.13E-04
Streptococcus vestibularis	1.08E-05	1.80E-04	4.15E-04	8.84E-04
motu linkage group 517	3.16E-04	2.10E-03	4.13E-04	1.77E-05
Enterobacter hormaechei/cloacae	1.62E-02	4.10E-02	3.95E-04	1.09E-05
motu linkage group 672	9.76E-03	2.97E-02	3.71E-04	2.02E-05
Streptococcus thermophilus	2.09E-05	2.33E-04	3.43E-04	9.39E-04
Eggerthella lenta	1.45E-06	3.97E-05	3.28E-04	8.81E-05
Streptococcus sp. C150	4.32E-04	2.67E-03	2.79E-04	2.98E-04
Lachnospiraceae bacterium 1 4 56FAA	6.83E-05	6.42E-04	2.67E-04	3.14E-05
Clostridium leptum	2.25E-03	9.83E-03	2.48E-04	1.03E-04
Holdemania filiformis	7.30E-07	2.44E-05	2.38E-04	5.38E-05
Anaerotruncus colihominis	4.08E-05	3.96E-04	2.20E-04	3.67E-05
Pseudoflavonifractor capillosus	7.99E-05	6.87E-04	1.79E-04	5.14E-05
Oxalobacter formigenes	4.82E-03	1.77E-02	1.75E-04	3.61E-05
motu linkage group 771	9.15E-03	2.87E-02	1.62E-04	1.44E-06
motu linkage group 696	1.13E-02	3.23E-02	1.53E-04	8.83E-05
Bifidobacterium breve	1.45E-02	3.73E-02	1.40E-04	2.60E-03
Eubacterium dolichum	2.53E-03	1.07E-02	1.35E-04	2.18E-06
Lactobacillus gasseri	5.80E-04	3.17E-03	1.07E-04	3.23E-03
Streptococcus infantis	4.82E-03	1.77E-02	9.30E-05	1.54E-04
Clostridium spiroforme	1.05E-02	3.13E-02	9.29E-05	4.59E-05
motu linkage group 691	1.15E-02	3.25E-02	9.06E-05	1.92E-05
Ruminococcaceae bacterium D16	1.32E-02	3.51E-02	8.77E-05	4.59E-05
Clostridium scindens	1.41E-03	6.86E-03	8.51E-05	2.71E-05
Streptococcus pneumoniae	1.83E-02	4.55E-02	7.51E-05	1.45E-04
Lactobacillus amylovorus	9.72E-03	2.97E-02	7.35E-05	1.26E-02
Acidaminococcus fermentans	1.78E-02	4.47E-02	6.23E-05	5.00E-04
Bifidobacterium dentium	5.14E-03	1.82E-02	5.83E-05	4.51E-04
Lactobacillus oris	2.11E-03	9.46E-03	4.40E-06	1.36E-03
Lactobacillus salivarius	1.10E-03	5.50E-03	4.36E-06	2.39E-03
Streptococcus sp. M143	1.45E-02	3.73E-02	1.65E-06	1.82E-05
Lactobacillus crispatus	5.10E-03	1.82E-02	9.42E-07	6.58E-04
Lactobacillus rhamnosus	1.10E-03	5.50E-03	0	2.36E-03
**Glipizide**				
**mOTU ID**	***P* value**	***q* value**	**Relative Abundance**	
			**Pre**	**Post**
Bacteroides eggerthii	3.82E-02	9.70E-01	1.56E-02	9.04E-03
Megasphaera elsdenii	1.43E-02	9.70E-01	6.43E-04	2.93E-04

motu linkage group 126	2.62E-02	9.70E-01	2.07E-03	4.38E-03
motu linkage group 151	3.72E-02	9.70E-01	5.46E-04	1.27E-03
motu linkage group 270	1.65E-02	9.70E-01	3.71E-03	8.80E-03

Supplementary Table 7 PERMANOVA analysis of microbial genes and plasma BA species

**Bile Acid**	**Sample Number**	**df**	**SumsOfSqs**	**MeanSqs**	**F.Model**	**R2**	**Pr(>F)**	***q* value**
**UDCA**	91	1	1.076	1.076	3.460	0.037	1.00E-04	5.00E-04
**GUDCA**	91	1	0.887	0.887	2.833	0.031	1.00E-04	5.00E-04
**LCA**	91	1	0.843	0.843	2.688	0.029	1.00E-04	5.00E-04
**DCA**	91	1	0.923	0.923	2.952	0.032	2.00E-04	7.50E-04
**GLCA**	91	1	0.544	0.544	1.715	0.019	6.90E-03	2.07E-02
**TUDCA**	91	1	0.497	0.497	1.565	0.017	2.14E-02	4.71E-02
**CDCA**	91	1	0.500	0.500	1.574	0.017	2.20E-02	4.71E-02
**GCA**	91	1	0.248	0.248	0.774	0.009	8.99E-01	9.76E-01
**TCDCA**	91	1	0.236	0.236	0.736	0.008	9.44E-01	9.76E-01
**GDCA**	91	1	0.431	0.431	1.355	0.015	6.96E-02	1.31E-01
**TCA**	91	1	0.224	0.224	0.700	0.008	9.76E-01	9.76E-01
**TLCA**	91	1	0.386	0.386	1.210	0.013	1.47E-01	2.45E-01
**TDCA**	91	1	0.310	0.310	0.970	0.011	4.86E-01	7.28E-01
**GCDCA**	91	1	0.281	0.281	0.877	0.010	6.91E-01	9.42E-01
**CA**	91	1	0.269	0.269	0.839	0.009	7.69E-01	9.62E-01

CA, cholic acid; GCA, glycocholic acid; TCA, taurocholic acid

CDCA, chenodeoxycholic acid; TCDCA, taurochenodeoxycholic acid; GCDCA, glycochenodeoxycholic acid

UDCA, ursodeoxycholic acid; TUDCA, tauroursodeoxycholic acid; GUDCA, glycoursodeoxycholic acid

DCA, deoxycholic acid; GDCA, glycodeoxycholic acid; TDCA, taurodeoxycholic acid

LCA, lithocholic acid; GLCA, glycolithocholic acid; TLCA; taurolithocholic acid

Supplementary Table 8 Annotation of bacterial genes involved in BA metabolism based on protein sequences associated with BA metabolism in the UniProt database

**Gene**	**Enzyme**	**KO**	**Annotation**	**Hit-keywords**	**Number of reference**	**Number of annotated gene**
baiG	NA	NA	Bile acid transporter	bile acid transporter	321	1284
bsh	3.5.1.24	K01442	Bile salt hydrolase	EC 3.5.1.24; K01442	375	1722
baiB	6.2.1.7	K15868	Bile acid-CoA ligase	EC 6.2.1.7; K15868	43	417
baiH	NA	K15873	NAD(H)-dependent 7β-hydroxy-3-oxo-Δ4-cholenoic acid oxidoreductase	K15873	1	323
baiA	1.1.1.395	K15869	3α-hydroxy bile acid-CoA-ester 3-dehydrogenase	EC 1.1.1.395; K15869	6	1867
baiCD	NA	K15870	NAD(H)-dependent 7α-hydroxy-3-oxo-Δ4-cholenoic acid oxidoreductase	K15870	2	429
baiF	3.1.2.26	K15871	Bile acid-CoA hydrolase	EC 3.1.2.26 (2.8.3.25); K15871	33	664
baiI	NA	K15874	Bile-acid 7β-dehydratase	K15874	1	20
baiE	4.2.1.106	K15872	Bile acid 7-α dehydratase	EC 4.2.1.106; K15872	43	68
7α-hsdh	1.1.1.159	K00076	7α-hydroxysteroid dehydrogenase	EC 1.1.1.159; K00076	184	9295
7β-hsdh	1.1.1.201	NA	7β-hydroxysteroid dehydrogenase	EC 1.1.1.201; 7β- hydroxysteroid dehydrogenase	2	13
**Total**					**1011**	**16102**

BlastP parameters for gene annotation: E value < 1E-5 and bit score > 60.

Supplementary Table 9 Comparison of the relative abundances of genes encoding enzymes involved in bacterial BA metabolism pre- and post-treatment in both arms

**Gene**	**Acarbose pre *vs.* post treatment**					**Glipizide pre *vs.* post treatment**				**Acarbose pre *vs.* Glipizide pre**			
	***P* value**	**Fold change**	**Enriched group**	**Median (relative abundance)**		***P* value**	**Fold change**	**Median (relative abundance)**		***P* value**	**Fold change**	**Median (relative abundance)**	
				**pre**	**post**			**pre**	**post**			**Acar**	**Glip**
baiG	6.66E-03	0.868	Post	2.61E-04	2.96E-04	4.91E-01	1.026	2.49E-04	2.43E-04	8.67E-01	1.024	2.61E-04	2.49E-04
bsh	4.51E-01	0.926	NS	3.12E-04	3.05E-04	6.54E-01	1.003	3.16E-04	3.14E-04	8.20E-01	1.021	3.12E-04	3.16E-04
baiB※	3.02E-04	1.392	Pre	1.10E-05	6.33E-06	4.69E-01	0.904	9.43E-06	1.01E-05	5.04E-01	1.050	1.10E-05	9.43E-06
baiH※	1.67E-01	0.617	NS	1.22E-05	1.21E-05	2.57E-01	0.884	1.11E-05	1.30E-05	7.16E-01	1.080	1.22E-05	1.11E-05
baiA※	1.06E-02	0.825	Pre	9.92E-04	1.01E-03	7.42E-01	0.970	1.71E-04	1.81E-04	3.24E-01	1.067	2.06E-04	1.71E-04
baiCD※	3.91E-01	0.712	NS	1.90E-05	2.01E-05	1.05E-01	0.896	1.76E-05	2.03E-05	2.39E-01	1.143	1.90E-05	1.76E-05
baiF※	2.30E-02	1.032	Pre	2.34E-05	1.94E-05	6.20E-01	0.963	2.39E-05	2.40E-05	9.21E-01	0.961	2.34E-05	2.39E-05
baiI	2.68E-03	1.555	Pre	1.10E-06	1.10E-07	3.91E-01	0.748	1.24E-06	8.76E-07	5.53E-01	0.774	1.10E-06	1.24E-06
baiE	1.09E-04	2.929	Pre	3.44E-06	1.15E-06	8.03E-01	1.562	3.78E-06	3.28E-06	6.76E-01	1.113	3.44E-06	3.78E-06
7α-hsdh※	6.66E-03	1.070	Pre	1.23E-03	1.13E-03	5.54E-01	0.997	1.16E-03	1.17E-03	9.65E-03	1.070	1.23E-03	1.16E-03
7β-hsdh	5.68E-05	0.236	Post	1.50E-06	2.63E-06	3.92E-01	0.903	9.68E-07	1.18E-06	3.39E-01	1.497	1.50E-06	9.68E-07

The symbol (※) indicates the genes with inconsistent annotations using the KOALA (KEGG Orthology And Links Annotation) pipeline.

NS, not significant ($P > 0.05$).

Supplementary Table 10 Faecal bile acids pre-and post-treatment in the Acarbose arm

**Faecal bile acid (ng/g)**	**Pre-Treatment**	**Post-Treatment**	***P* value (Absolute value)**	**Pre-Treatment (% of ABA)**	**Post-Treatment (% of ABA)**	***P* value (% of ABA)**
**Primary BAs**						
CA	781.42±1110.84	752.1±881.29	7.96E-01	9.79±13.34	18.64±20.13	1.33E-03
CDCA	638.36±933.5	603.98±868.9	4.22E-01	8.12±9.79	13.07±12.89	2.97E-04
GCA	18.34±40.24	11.09±6.07	7.98E-02	0.34±0.61	0.42±0.33	4.75E-03
TCA	22.84±43.4	17.24±24.42	5.72E-01	0.37±0.5	0.56±0.46	4.19E-04
TCDCA	21.46±23.62	15.77±6.4	3.75E-01	0.33±0.32	0.3±0.1	4.32E-01
GCDCA	22.73±53.09	13.34±13.77	7.50E-02	0.4±0.79	0.45±0.33	1.48E-02
**Secondary BAs**						
UDCA	1270.29±2079.42	954.03±1644.08	9.64E-01	14.14±18.06	19.26±18.33	3.39E-02
DCA	1553.16±934.29	724.44±787.64	1.47E-05	32.62±16.98	26.04±19.77	1.04E-02
LCA	1333.92±1010.54	539.54±637.42	1.98E-06	28.64±18.58	19.85±20.08	4.05E-03
GDCA	24.38±26.61	15.87±3.59	3.88E-04	0.51±0.45	0.65±0.46	3.31E-02
7KLCA	111.49±156.93	51.33±55.78	2.66E-02	1.33±1.27	1.22±0.95	3.79E-01
12KLCA	259.35±232.37	131.77±149.32	2.14E-04	5.26±3.7	4.53±3.31	1.18E-01
**BA index**						
UnconBA/ConBA	74.18±38.18	62.65±40.97	5.53E-02			
12α-OH/non 12α-OHBA	0.80±0.28	0.87±0.45	5.32E-01			
PBA/SBA	0.40±0.66	1.46±2.9	5.20E-04			
ABA	5962.77±3413.34	3740.42±2527.12	4.19E-05			

Data are presented as mean ± SD; 100 samples had faecal BA profile determined. Unit of all BAs is ng/g dry faces

7KLCA, 7-ketolithocholic acid; 12KLCA, 12-ketolithocholic acid

UnconBA/ConBA = (CA + UDCA + CDCA + DCA + LCA+7KLCA+12KLCA) / (GCDCA + GDCA + GCA + TCA + TCDCA)

12 α-OH/non12α-OHBA =（GCA+CA+TCA+GDCA+DCA) / (CDCA+TCDCA+LCA+UDCA+GCDCA+7KLCA+12KLCA）

PBA/SBA = (GCDCA+ TCDCA + CDCA + CA + TCA + GCA) / (UDCA + DCA + GDCA + LCA +7KLCA+12KLCA)

ABA=GCDCA+ TCDCA + CDCA + CA + TCA + GCA + UDCA + DCA + GDCA + LCA +7KLCA+12KLCA
P value (Absolute value) indicates that the *P* values were calculated based on absolute values of faecal BAs.
P value (% of ABA) indicates that the *P* values were calculated based on percentage of each BA species in ABA.

Supplementary Table 11 Comparison of baseline clinical indices in T2D patients stratified according to the two distinct intestinal microbiome clusters: Cluster B *vs.* Cluster P

**Clinical index**	**Unit**	***P* value**	**Cluster B**	**Cluster P**
Age	Years	6.39E-01	53.3 ± 6.6	53.7 ± 7.4
Sex	Male/Total	7.25E-01	37/68	21/26
BMI	kg/m^2	4.27E-01	26.2 ± 3.5	26.2 ± 2.7
BW	kg	6.33E-01	72.8 ± 9.9	74.4 ± 12.1
SBP	mmHg	5.23E-02	127 ± 16	135 ± 20
DBP	mmHg	5.69E-01	81 ± 9	83 ± 11
Waist circumference	cm	3.82E-01	90.6 ± 7.7	92.5 ± 10.1
WHR		7.30E-02	0.9 ± 0.1	0.9 ± 0.1
HbA1c	%	3.95E-01	7.7 ± 0.9	7.5 ± 0.7
Blood Glucose level 0'	mmol/L	9.17E-02	7.9 ± 1.4	7.3 ± 1.1
Blood Glucose level 30'	mmol/L	1.30E-01	10.9 ± 2	10.3 ± 1.9
Blood Glucose level 60'	mmol/L	2.57E-01	14.2 ± 2.4	13.8 ± 1.8
Blood Glucose level 120'	mmol/L	2.43E-01	14.8 ± 2.9	14 ± 2.4
Blood Glucose level 180'	mmol/L	7.42E-01	12.1 ± 3.2	11.5 ± 3
AUCGlu	mmol/L*min	2.28E-01	2336 ± 377	2223 ± 310
C Peptide 0'	ng/mL	3.85E-01	1.2 ± 0.4	1.1 ± 0.5
C Peptide 30'	ng/mL	8.14E-01	1.7 ± 0.7	1.7 ± 0.7
C Peptide 60'	ng/mL	8.05E-01	2.6 ± 1.2	2.5 ± 1.2
C Peptide 120'	ng/mL	8.52E-01	3.9 ± 1.6	3.8 ± 1.4
C Peptide 180'	ng/mL	5.88E-01	3.6 ± 1.5	3.3 ± 1.1
AUCCpep	ng/mL*min	8.14E-01	518 ± 189	509 ± 182
Insulin 0'	pmol/L	4.69E-01	82.5 ± 40.5	82.1 ± 58.4
Insulin 30'	pmol/L	8.11E-01	136.4 ± 67.8	139.1 ± 87.1
Insulin 60'	pmol/L	8.39E-01	240.4 ± 129.7	252.6 ± 182.7
Insulin120'	pmol/L	7.42E-01	333.2 ± 171.7	363.3 ± 248.7
Insulin180'	pmol/L	7.55E-01	282.6 ± 163.2	282.2 ± 179.1
AUCIns	pmol/L*min	7.29E-01	969.8 ± 452.5	1036.3 ± 652.1
Ins30/G30*	uIU/mmol	4.80E-01	2 ± 3	2 ± 2
HOMA β*	%	6.72E-01	51 ± 30	58 ± 44
HOMA IR*	uIU*mmol	2.07E-01	3.8 ± 2.1	3.4 ± 2.5
TC	mmol/L	7.64E-01	5 ± 0.9	5.1 ± 1.5
TG	mmol/L	4.79E-01	2.3 ± 1.6	2.3 ± 2.3
LDL	mmol/L	3.41E-01	1.2 ± 0.5	1.2 ± 0.2
HDL	mmol/L	9.74E-01	3.1 ± 0.8	3.2 ± 1.1
APOA	g/L	9.94E-01	1.3 ± 0.2	1.4 ± 0.2
APOB	g/L	9.24E-01	1 ± 0.2	1.1 ± 0.3
AST	IU/L	8.21E-01	26.8 ± 12.4	32.5 ± 33.8
AKP	IU/L	3.73E-03	68 ± 17.8	81.7 ± 21.7
ALB	g/L	4.39E-01	46.8 ± 26.7	44.4 ± 4.2
ALT	IU/L	7.71E-01	34.7 ± 20.2	39.3 ± 38.6

γGT	IU/L	2.20E-01	38.9 ± 37.5	48.1 ± 54.4
FLI		4.50E-01	284 ± 487	309 ± 435
Uric acid	umol/L	3.89E-01	5 ± 1.2	5.2 ± 1
Bun	mmol/L	9.38E-01	301.2 ± 73.9	301.3 ± 65.5
creatine	umol/L	4.24E-01	66.6 ± 14.8	69.2 ± 12.4
IFNr1	pg/mL	1.82E-01	45.6 ± 42.6	34.8 ± 17.1
IL6	pg/mL	4.44E-02	3 ± 5	3 ± 4
IL8	ng/mL	8.13E-01	17 ± 26	19 ± 25
LPS binding protein	ng/mL	1.85E-01	20.6 ± 7.3	18.7 ± 8
MCP1	pg/mL	4.54E-01	244 ± 136	246 ± 78
mpo	ng/mL	2.74E-01	386 ± 352	465 ± 363
PAI1	pg/mL	6.36E-01	57833 ± 21243	59240 ± 19940
TNF α	pg/mL	2.93E-01	4.6 ± 2.2	5.7 ± 4.5
CRP	ug/mL	3.10E-01	12 ± 16	21 ± 49
Endothelin-1	MFI	5.61E-01	12.8 ± 2.7	12.9 ± 3.4
FetuinA	ug/mL	7.90E-01	333.8 ± 50.2	332.6 ± 40.5
FGF19	MFI	8.03E-01	23 ± 4.1	23.3 ± 3.8
FGF21	MFI	5.37E-01	138.6 ± 60.8	156 ± 136
FGF23	MFI	1.32E-01	45.9 ± 9.3	42 ± 6.8
Adiponectin	pg/mL	6.33E-01	5307140 ± 3052570	6121582 ± 4297114
Leptin	pg/mL	7.32E-01	6491 ± 6637	6502 ± 5786

Data are presented as mean ± SD; * the Unit of insulin is converted to uIU/L

MFI, mean fluorescence intensity determined by using the Luminex 200™ instrument

Supplementary Table 12 Comparison of baseline plasma bile acid profiles in T2D patients with two distinct intestinal microbiome compositions: Cluster B *vs.* Cluster P

**Plasma bile acid (nmol/L)**	***P* value**	**Fold change**	**Median (nM)**		**Mean±SD (nM)**	
			**Cluster B**	**Cluster P**	**Cluster B**	**Cluster P**
DCA	5.16E-03	0.543	113.48	208.88	143 ± 131	316 ± 294
GDCA	1.91E-02	0.531	177.02	333.18	372 ± 745	725 ± 1053
UDCA	2.84E-02	1.605	104.485	65.1	244 ± 350	98.9 ± 91.3
LCA	7.53E-03	0.786	11.4	14.51	12.5 ± 9.8	26.4 ± 25.8
GLCA	2.10E-02	0.518	3.525	6.81	7.1 ± 9.2	17.9 ± 35.5
GCDCA	9.68E-01	1.117	732.865	656.31	1063 ± 1329	1339 ± 1596
GUDCA	1.59E-01	1.325	66.645	50.29	137 ± 173	71.4 ± 67.2
CA	3.62E-01	1.099	47.82	43.52	117 ± 214	112 ± 186
GCA	7.25E-01	0.979	176.155	180.01	356 ± 673	450 ± 775
TUDCA	8.06E-02	1.608	5.9	3.67	17.7 ± 28.8	9.2 ± 16.1
TCA	1.68E-01	2.211	15.455	6.99	37.5 ± 71	38 ± 105
CDCA	4.16E-01	1.023	166.85	163.03	322 ± 402	220 ± 195
TCDCA	4.80E-01	1.097	57.84	52.72	106 ± 152	109 ± 210
TDCA	2.02E-01	0.733	12.985	17.72	31.6 ± 59	40.7 ± 79.9
TLCA	1.23E-01	0.625	3.56	5.7	6.4 ± 10.2	11.1 ± 22.7
ABA	7.79E-01	0.843	2083.12	2471.62	3222 ± 3412	3859 ± 4121
SBA	3.30E-01	0.722	673.03	932.42	971 ± 1013	1317 ± 1383
PBA	8.00E-01	0.988	1238.8	1254.17	2002 ± 2401	2268 ± 2735
PBA/SBA	3.79E-02	1.428	1.89	1.32	2.2 ± 1.2	1.8 ± 1.6
UnconBA/ConBA	7.79E-01	0.922	0.40	0.44	0.6 ± 0.5	0.6 ± 0.5
12α-OH/non 12α-OHBA	8.17E-03	0.720	0.62	0.86	0.6 ± 0.4	0.9 ± 0.6
ConBA	7.86E-01	1.121	1292.835	1153.3	2135 ± 2999	2812 ± 3798
BconBA	3.04E-01	0.748	315.12	421.56	571 ± 887	875 ± 1217
PconBA	8.83E-01	1.136	1021.18	899.06	1563 ± 2178	1936 ± 2623
UnconBA	7.79E-01	0.846	498.99	589.89	838 ± 859	773 ± 591
BunconBA	5.31E-01	0.928	285.64	307.87	400 ± 342	442 ± 328
PunconBA	4.91E-01	0.980	209.125	213.46	439 ± 581	332 ± 340
GlycinBA	7.59E-01	1.091	1192.74	1092.79	1935 ± 2724	2603 ± 3417
TaurineBA	4.63E-01	0.905	102.655	113.44	199 ± 293	208 ± 429

ABA=UDCA + GCDCA + GDCA + GUDCA + CA + GCA + TUDCA + TCA + CDCA + DCA + TCDCA + TDCA + GLCA + LCA + TLCA
SBA=UDCA + GUDCA + TUDCA + DCA + TDCA + GDCA + LCA + GLCA + TLCA
PBA=GCDCA+ TCDCA + CDCA + CA + TCA + GCA
PBA/SBA = GCDCA+ TCDCA + CDCA + CA + TCA + GCA)/(UDCA + GUDCA + TUDCA + DCA + TDCA + GDCA + LCA + GLCA + TLCA
UnconBA/ConBA= CA + UDCA + CDCA + DCA + LCA)/(GCDCA + GDCA + GUDCA + GCA + TUDCA + TCA + TCDCA + TDCA + GLCA + TLCA
12 α-OH/non12α-OHBA = GCA+CA+TCA+GDCA+DCA+TDCA)/(TUDCA+CDCA+TCDCA+GLCA+LCA+UDCA+GCDCA+TLCA+GUDCA
ConBA=GCDCA + GDCA + GUDCA + GCA + TUDCA + TCA + TCDCA + TDCA + GLCA + TLCA
BconBA= GUDCA + TUDCA + TDCA + GDCA + GLCA + TLCA
PconBA=GCDCA+ TCDCA + TCA + GCA
UnconBA=CA + UDCA + CDCA + DCA + LCA
BunconBA=UDCA + DCA + LCA
PunconBA=CDCA + CA
GlycinBA=GCDCA + GDCA + GUDCA + GCA + GLCA
TaurineBA = TUDCA + TCA + TCDCA + TDCA + TLCA

Supplementary Table 13 Comparison of baseline faecal bile acid profiles in T2D patients in the Acarbose arm with two distinct intestinal microbiome compositions: Cluster B *vs.* Cluster P

**Fecal bile acid (ng/g)**	**Acarbose arm**					
	**Median (Absolute value)**			**Median (% of ABA)**		
	**Cluster B**	**Cluster P**	***P* value**	**Cluster B**	**Cluster P**	***P* value**
**Primary BAs**						
CA	208.00	21.87	3.69E-02	4.66	0.45	2.42E-02
CDCA	351.99	56.78	8.92E-02	6.52	1.07	5.18E-02
GCA	9.67	8.20	1.74E-01	0.21	0.21	9.53E-01
TCA	11.29	11.87	9.37E-01	0.28	0.26	9.55E-01
TCDCA	12.86	11.76	2.61E-01	0.19	0.29	6.93E-01
GCDCA	10.95	9.39	4.25E-01	0.22	0.23	7.79E-01
**Secondary BAs**						
UDCA	326.21	43.34	9.66E-02	7.37	1.00	2.37E-02
DCA	1552.04	1757.16	1.95E-01	33.48	42.88	7.11E-02
LCA	991.54	1698.36	2.00E-02	26.91	43.84	1.35E-02
GDCA	17.97	18.42	6.36E-01	0.42	0.48	3.89E-01
7KLCA	86.82	14.43	5.42E-02	1.01	0.40	2.72E-02
12KLCA	177.06	248.26	3.88E-01	4.51	5.75	3.88E-01
**BA index**						
UnconBA/ConBA	68.77	82.04	5.08E-01			
12α-OH/non 12α-OHBA	0.74	0.85	9.58E-02			
PBA/SBA	0.17	0.02	2.42E-02			
ABA	6157.32	4542.35	1.10E-01			

Supplementary Table 14 Comparison of baseline relative abundance profiles of genes encoding enzymes involved in BA metabolism in T2D patients stratified according to the two distinct intestinal microbiome enterotype-like clusters: Cluster B *vs.* Cluster P

**Gene**	**94 baseline samples from 2 Clusters**					
	***P* value**	***q* value**	**Fold change**	**Enriched group**	**Median (Cluster B)**	**Median (Cluster P)**
baiG	3.69E-07	5.43E-06	0.705	Cluster P	2.33E-04	3.25E-04
bsh	7.39E-13	9.76E-11	1.572	Cluster B	3.29E-04	2.13E-04
baiB※	9.09E-01	9.09E-01	0.961	NS	1.09E-05	1.09E-05
baiA※	8.74E-11	2.88E-09	0.647	Cluster P	1.65E-04	2.61E-04
baiCD※	2.74E-04	2.26E-03	1.503	Cluster B	2.10E-05	1.50E-05
baiH※	7.33E-04	3.87E-03	1.436	Cluster B	1.33E-05	8.60E-06
baiF※	1.80E-01	2.16E-01	0.817	NS	2.34E-05	2.85E-05
baiE	1.59E-01	2.16E-01	0.943	NS	3.06E-06	3.55E-06
baiI	9.80E-02	1.80E-01	0.806	NS	7.85E-07	1.42E-06
7α-hsdh※	2.54E-02	5.98E-02	1.063	NS	1.21E-03	1.14E-03
7β-hsdh	6.58E-03	2.07E-02	2.417	Cluster B	1.41E-06	6.70E-07

The symbol (※) indicates the genes with inconsistent annotations by using the KOALA (KEGG Orthology And Links Annotation) pipeline.
NS, not significant ($P > 0.05$).

Supplementary Table 15 Comparison of clinical parameters altered by Acarbose and Glipizide treatments in patients stratified according to baseline clustering of the gut microbiome

Clinical index	unit	Acarbose					
		Cluster B			Cluster P		
		Pre	Post	*P* value	Pre	Post	*P* value
Age	years	53.6 ± 6.3	53.6 ± 6.3	1.00E+00	51.5 ± 7.9	51.5 ± 7.9	1.00E+00
BMI	kg/m^2	26.4 ± 3.4	25.5 ± 3.5	3.26E-05	26.1 ± 2.9	25.1 ± 2.4	3.49E-03
BW	kg	74.5 ± 9.3	71.9 ± 9.3	2.32E-05	75.4 ± 12.7	72.6 ± 12.2	3.67E-03
Waist circumference	cm	91.1 ± 7.4	88.5 ± 7.2	4.81E-03	92.3 ± 10.9	88.9 ± 8	3.00E-02
WHR		0.9 ± 0	0.9 ± 0	3.63E-02	0.9 ± 0.1	0.9 ± 0.1	6.83E-02
SBP	mmHg	127 ± 16.2	120.9 ± 14.3	1.23E-01	127.8 ± 15.2	119.8 ± 9.4	6.45E-02
DBP	mmHg	82.1 ± 8.3	75.4 ± 8.6	1.95E-04	80.5 ± 8.8	74.9 ± 7	4.91E-02
Blood Glucose level 0'	mmol/L	7.9 ± 1.4	6.6 ± 0.9	2.34E-06	7 ± 0.8	6.7 ± 0.9	3.03E-01
Blood Glucose level 30'	mmol/L	10.7 ± 2.1	7.6 ± 1.5	4.07E-10	9.7 ± 1.6	7.6 ± 1.1	1.22E-04
Blood Glucose level 60'	mmol/L	14 ± 2.5	9.1 ± 2	5.82E-11	13.1 ± 1.6	9.1 ± 1.1	1.22E-04
Blood Glucose level 120'	mmol/L	14.6 ± 2.9	9.2 ± 2	1.75E-07	13.3 ± 2.1	9.2 ± 1.4	6.10E-05
Blood Glucose level 180'	mmol/L	11.7 ± 3	7.9 ± 2.2	7.36E-09	10.7 ± 2.9	7.8 ± 1.7	2.44E-04
HbA1c	%	7.6 ± 0.9	6.4 ± 0.5	3.54E-07	7.3 ± 0.6	6.3 ± 0.5	1.09E-03
TC	mmol/L	5 ± 1	4.7 ± 1	5.94E-02	5.5 ± 1.6	4.9 ± 1.6	2.69E-02
TG	mmol/L	2.5 ± 1.8	1.5 ± 1.1	6.66E-04	2.5 ± 3	1.7 ± 1.4	1.67E-02
HDL	mmol/L	3 ± 0.8	3 ± 0.8	8.88E-01	3.3 ± 1.4	2.9 ± 1.3	1.10E-01
LDL	mmol/L	1.1 ± 0.4	1.2 ± 0.4	1.22E-01	1.2 ± 0.2	1.2 ± 0.2	5.56E-01
APOA	g/L	1.3 ± 0.2	1.2 ± 0.1	1.13E-02	1.4 ± 0.2	1.3 ± 0.2	1.25E-01
APOB	g/L	1 ± 0.2	1 ± 0.2	4.24E-01	1.1 ± 0.4	0.9 ± 0.4	1.88E-01
Insulin 0'	pmol/L	85.1 ± 40	67.3 ± 48.9	9.63E-03	80.8 ± 56.8	71.6 ± 30.3	7.15E-01
Insulin30'	pmol/L	140.9 ± 76.3	96.8 ± 63.4	3.01E-03	134.3 ± 90.2	110.2 ± 41.5	1.73E-01
Insulin 60'	pmol/L	253 ± 137.4	153.8 ± 106.2	2.83E-04	271.2 ± 217.1	166.9 ± 72.8	3.53E-02
Insulin120'	pmol/L	346.5 ± 191.3	201.3 ± 118.6	3.16E-05	396.7 ± 289.6	236.4 ± 121.1	5.74E-02
Insulin180'	pmol/L	277.9 ± 164.8	157.9 ± 134	7.24E-06	306.7 ± 217.5	198.5 ± 105.6	6.76E-02
AUC Ins	pmol/L*min	995.1 ± 482.8	608.9 ± 361.6	1.61E-05	1111.2 ± 762.5	715.3 ± 281.2	8.03E-02
C Peptide 0'	ng/mL	1.3 ± 0.5	1.1 ± 0.5	3.28E-03	1.1 ± 0.5	1.1 ± 0.4	5.83E-01

C Peptide 30'	ng/mL	1.8 ± 0.9	1.4 ± 0.6	4.56E-05	1.6 ± 0.8	1.4 ± 0.5	1.34E-02
C Peptide 60'	ng/mL	2.8 ± 1.3	1.9 ± 0.9	1.23E-04	2.5 ± 1.4	1.9 ± 0.9	6.10E-04
C Peptide 120'	ng/mL	4 ± 1.7	2.8 ± 1.2	3.16E-05	3.8 ± 1.5	2.7 ± 1	1.22E-04
C Peptide 180'	ng/mL	3.7 ± 1.7	2.5 ± 1.2	1.50E-06	3.3 ± 1.3	2.5 ± 1.3	2.45E-02
AUCGlu	mM*min	2299 ± 388	1525 ± 263	2.91E-11	2102 ± 254	1464 ± 270	6.10E-05
AUCCpep	ng/mL*min	537 ± 215	384 ± 146	9.67E-06	507 ± 205	383 ± 139	3.66E-04
HOMA IR*	uIU*mmol	4 ± 2.3	2.6 ± 2	1.03E-03	3.2 ± 2.2	2.7 ± 1.2	7.15E-01
HOMA β*	%	51.4 ± 23.5	58 ± 43.6	7.65E-01	60 ± 46.1	65 ± 45.6	5.42E-01
ΔIns30/G30*	uIU/mmol	2.1 ± 3.7	33 ± 115	1.24E-02	2.6 ± 2.9	27 ± 102	6.35E-01
Bun	mmol/L	309.3 ± 76.8	329.2 ± 58.9	1.71E-02	306.2 ± 67.2	326.5 ± 65.9	2.21E-01
creatine	umol/L	67.1 ± 15.8	67.6 ± 16	5.31E-01	68 ± 10.6	67.4 ± 10.4	1.00E+00
Uric acid	umol/L	4.9 ± 1	4.7 ± 1.4	2.06E-01	5.1 ± 1.1	5 ± 1.2	7.01E-01
FLI		315 ± 570	103 ± 167	1.76E-06	387 ± 529	94.9 ± 82.8	9.77E-04
AKP	IU/L	68.4 ± 20.9	59.2 ± 18.9	2.98E-04	85.4 ± 24.7	77.5 ± 24.9	1.49E-01
ALB	g/L	49.8 ± 37.5	42.3 ± 2.6	5.29E-02	45.6 ± 4.8	44.4 ± 3.7	1.26E-01
ALT	IU/L	37.7 ± 21.9	29.4 ± 21.1	2.39E-02	40.7 ± 37.8	44.4 ± 61.1	3.15E-01
γGT	IU/L	42.8 ± 47	24.8 ± 16.9	5.29E-05	60.1 ± 67.7	43.1 ± 56.1	2.03E-02
AST	IU/L	28.2 ± 12.3	22.5 ± 9.8	9.26E-03	35.9 ± 41.6	29.4 ± 24	1.84E-01
LPS binding protein	ng/mL	19.6 ± 5.8	17.4 ± 6.6	3.17E-01	15.4 ± 5	17.6 ± 5.9	3.91E-01
Adiponectin	pg/ml	4659142 ± 3197330	5157340 ± 2775458	9.07E-03	6825516 ± 5265272	10063264 ± 10737091	7.30E-02
Leptin	pg/mL	6319 ± 6148	6481 ± 9720	1.58E-01	7201 ± 6297	4522 ± 2763	6.71E-03
CRP	ug/mL	13.6 ± 17.5	30 ± 75.1	8.53E-01	10.2 ± 8.4	19.6 ± 27.3	2.29E-01
Endothelin-1	pg/mL	12.4 ± 2.4	13.4 ± 2.8	3.01E-02	13.7 ± 4.1	14.3 ± 2.8	2.08E-01
MCP1	pg/mL	245.5 ± 93.8	256.6 ± 91.8	6.96E-02	212 ± 75.3	252.8 ± 89.7	6.71E-03
mpo	ng/mL	366 ± 358	276 ± 223	5.43E-01	411 ± 344	467 ± 409	1.00E+00
PAI1	pg/mL	56210 ± 19392	52770 ± 20498	7.10E-01	54085 ± 15520	61007 ± 26493	2.52E-01
TNF α	pg/mL	4.7 ± 2.1	4.8 ± 2.4	8.78E-01	4 ± 2	4.6 ± 1.9	7.82E-02
IFNr1	pg/mL	38.8 ± 23.1	42.1 ± 24	2.59E-01	37.6 ± 20.2	38.8 ± 12.9	6.09E-01
IL6	pg/mL	2.6 ± 5.2	4.8 ± 9.8	3.46E-01	1.6 ± 0.8	2.7 ± 2.4	1.29E-01
IL8	ng/mL	15.1 ± 21.8	11.9 ± 17.8	6.48E-01	9.6 ± 9.6	15.4 ± 24.9	4.63E-01
FGF19	MFI	22.4 ± 4.3	19.7 ± 4.7	7.21E-04	22.6 ± 3.7	19.3 ± 3	6.27E-03
FGF21	MFI	143 ± 64.9	100.1 ± 65.8	2.00E-04	136.4 ± 79.7	97.9 ± 41.5	2.62E-03

FGF23	MFI	44.9 ± 6.7	37.1 ± 10.2	5.55E-05	42.4 ± 6.6	43.5 ± 6.4	6.29E-01
CA	nmol/L	109.2±145	214±260	0.018677421	76.7±95.3	204.9±263.5	0.049438477
CDCA	nmol/L	320.6±365	687.7±527.3	0.000781945	238.4±208	659±623.3	0.013427734
GCA	nmol/L	302.1±429.9	272.7±499.1	0.02732318	525.3±968.6	191.3±179.2	0.104003906
GDCA	nmol/L	331.2±539	134.2±298.9	0.006609388	810.7±1210.2	276.3±255.6	0.104003906
TCA	nmol/L	34±54.1	25.1±53.1	0.00432581	56.3±138.2	13.2±10.2	0.153076172
TDCA	nmol/L	31.7±59.5	10±14.2	0.001115285	55±105	19±17	0.153076172
TLCA	nmol/L	5.4±7.4	3.2±4.1	0.023753465	14.7±30	5.7±4.1	0.71484375
TUDCA	nmol/L	17.5±29.5	8±8.6	0.041045422	13±20.8	6.9±6.8	0.426269531
**Clinical index**	**Unit**	**Glipizide**					
		**Cluster B**			**Cluster P**		
		**Pre**	**Post**	***P* value**	**Pre**	**Post**	***P* value**
Age	Years	53 ± 7	53 ± 7	1.00E+00	56.8 ± 5.6	56.8 ± 5.6	1.00E+00
BMI	kg/m^2	25.9 ± 3.6	25.7 ± 3.6	2.84E-01	26.4 ± 2.7	25.7 ± 2.3	3.20E-01
BW	kg	70.9 ± 10.4	70.3 ± 10.2	2.94E-01	73.2 ± 11.8	71.3 ± 10.8	3.48E-01
Waist circumference	cm	90 ± 8.1	89.3 ± 8.2	4.45E-01	92.9 ± 9.2	90 ± 9.4	1.71E-01
WHR		0.9 ± 0.1	0.9 ± 0	6.88E-02	0.9 ± 0	0.9 ± 0.1	1.41E-01
SBP	mmHg	126.3 ± 15.3	125 ± 14.8	3.46E-01	144.6 ± 21.8	126.5 ± 14.2	5.37E-02
DBP	mmHg	79.5 ± 8.6	76.3 ± 8.7	7.08E-02	85.4 ± 13.6	75.6 ± 10.7	9.10E-02
Blood Glucose level 0'	mmol/L	7.9 ± 1.4	6.7 ± 1.3	5.16E-05	7.7 ± 1.4	6.6 ± 0.9	1.44E-02
Blood Glucose level 30'	mmol/L	11.1 ± 1.8	9 ± 2.4	1.04E-04	11.1 ± 2	8.8 ± 1.8	9.77E-03
Blood Glucose level 60'	mmol/L	14.5 ± 2.4	11.5 ± 2.6	8.10E-07	14.7 ± 1.9	11.5 ± 1.8	4.88E-03
Blood Glucose level 120'	mmol/L	14.9 ± 2.9	10.9 ± 2.7	2.08E-07	15 ± 2.5	10.6 ± 3.1	1.95E-03
Blood Glucose level 180'	mmol/L	12.6 ± 3.4	8.8 ± 3	3.55E-07	12.7 ± 3	9.2 ± 3.4	9.77E-04
HbA1c	%	7.7 ± 1	6.3 ± 0.7	1.33E-06	7.7 ± 0.8	6.2 ± 0.7	3.84E-03
TC	mmol/L	5.1 ± 0.9	4.9 ± 0.8	4.77E-01	4.5 ± 1.3	4.5 ± 0.8	3.20E-01
TG	mmol/L	2.1 ± 1.2	1.8 ± 0.9	7.05E-01	2 ± 1.2	1.5 ± 0.6	3.65E-01
HDL	mmol/L	3.1 ± 0.8	2.9 ± 0.8	1.25E-01	3 ± 0.5	2.7 ± 0.7	1.23E-01
LDL	mmol/L	1.2 ± 0.5	1.3 ± 0.7	7.76E-01	1.2 ± 0.2	1.3 ± 0.3	9.97E-02
APOA	g/L	1.4 ± 0.2	1.3 ± 0.2	4.94E-01	1.3 ± 0.1	1.2 ± 0.2	2.50E-01
APOB	g/L	1 ± 0.2	1 ± 0.2	5.88E-01	1 ± 0.1	0.9 ± 0.1	2.50E-01
Insulin 0'	pmol/L	79.6 ± 41.3	77.1 ± 44.4	9.34E-01	83.9 ± 63	73.9 ± 61.5	4.65E-01

Insulin30'	pmol/L	131.6 ± 58	197.3 ± 114.3	4.89E-05	145.9 ± 86.8	184.8 ± 111.9	1.75E-01
Insulin 60'	pmol/L	226.3 ± 120.9	290.7 ± 183	6.22E-03	227.3 ± 127.8	261.7 ± 142.9	4.13E-01
Insulin120'	pmol/L	319.1 ± 149.8	391.6 ± 238.2	5.29E-02	318.1 ± 181.8	342.2 ± 179.3	7.00E-01
Insulin180'	pmol/L	287.6 ± 164.1	318.9 ± 158.3	1.44E-01	249.2 ± 109.2	282.6 ± 207.5	5.77E-01
AUC Ins	pmol/L*min	942.3 ± 422.6	1172.2 ± 630.4	1.25E-02	934.2 ± 478.9	1044.9 ± 558.6	5.77E-01
C Peptide 0'	ng/mL	1.1 ± 0.3	1.1 ± 0.3	9.34E-01	1.2 ± 0.5	1.1 ± 0.6	2.06E-01
C Peptide 30'	ng/mL	1.5 ± 0.5	2.1 ± 0.6	1.73E-06	1.8 ± 0.6	2.1 ± 0.9	1.75E-01
C Peptide 60'	ng/mL	2.4 ± 0.9	2.9 ± 0.8	2.61E-04	2.5 ± 0.9	2.9 ± 1.3	4.13E-01
C Peptide 120'	ng/mL	3.8 ± 1.3	4.2 ± 1.5	5.21E-02	3.8 ± 1.3	4.1 ± 1.4	2.40E-01
C Peptide 180'	ng/mL	3.6 ± 1.3	3.8 ± 1.1	3.32E-01	3.3 ± 0.9	3.9 ± 1.5	9.98E-02
AUCGlu	mmol/L*min	2379 ± 368	1808 ± 373	4.10E-08	2388 ± 315	1793 ± 333	1.95E-03
AUCCpep	ng/mL*min	497 ± 156	575 ± 152	6.48E-03	512 ± 154	575 ± 195	2.40E-01
HOMA IR*	uIU*mmol	3.5 ± 1.8	3 ± 1.9	1.06E-01	3.7 ± 2.8	2.9 ± 2.6	1.47E-01
HOMA β*	%	51.5 ± 36.2	74.5 ± 67.5	9.34E-03	55.2 ± 43.1	60.4 ± 42	7.00E-01
ΔIns30/G30*	uIU/mmol	2 ± 1.9	11.6 ± 17.3	1.29E-06	2.2 ± 1.7	9.2 ± 9.4	9.77E-03
Bun	mmol/L	292 ± 70.6	290.7 ± 49.9	7.19E-01	293.9 ± 65.5	305.6 ± 53.8	2.75E-01
creatine	umol/L	66 ± 13.7	64.7 ± 11.9	9.04E-01	70.9 ± 15.2	70.5 ± 15.8	5.75E-01
Uric acid	umol/L	5.1 ± 1.4	5.1 ± 1.5	8.82E-01	5.5 ± 1	5.2 ± 1.4	9.59E-01
FLI		247 ± 367	174 ± 289	1.35E-01	208 ± 266	129 ± 226	8.40E-02
AKP	IU/L	67.6 ± 14.1	64.3 ± 14.4	1.84E-01	76.1 ± 15.7	71.3 ± 22.4	3.65E-02
ALB	g/L	43.8 ± 3	43.6 ± 3.4	7.84E-01	42.7 ± 2.6	42.8 ± 3.3	6.08E-01
ALT	IU/L	31.5 ± 17.8	25.3 ± 12.3	5.72E-02	37.5 ± 41.5	20.4 ± 6	2.84E-01
γGT	IU/L	34.5 ± 22.4	29.7 ± 24.2	9.75E-02	30.2 ± 12.6	22.6 ± 18	1.05E-01
AST	IU/L	25.2 ± 12.5	21.2 ± 5.8	1.12E-01	27.7 ± 19.9	19.6 ± 3.1	2.33E-01
LPS binding protein	ng/mL	21.8 ± 8.7	18.1 ± 4.7	2.12E-02	22.8 ± 9.3	20.6 ± 9.3	7.65E-01
Adiponectin	pg/mL	6036138 ± 2749742	6253072 ± 3047519	4.10E-01	5161672 ± 2365119	4741547 ± 2665058	7.65E-01
Leptin	pg/mL	6690 ± 7262	7575 ± 7318	3.21E-02	5614 ± 5218	3316 ± 1930	1.47E-01
CRP	ug/mL	10.1 ± 13.9	10.3 ± 8.1	2.95E-01	36.2 ± 74.8	18.6 ± 20.1	7.65E-01
Endothelin-1	pg/mL	13.3 ± 3	13.7 ± 3.8	4.10E-01	11.7 ± 1.6	12.2 ± 2.1	5.38E-01
MCP1	pg/mL	243 ± 174	255 ± 174	1.60E-01	293.3 ± 55	287.1 ± 86.3	7.00E-01
mpo	ng/mL	407 ± 349	342 ± 417	4.75E-02	537 ± 393	340 ± 215	3.20E-01
PAI1	pg/mL	59660 ± 23329	56888 ± 22053	3.60E-01	66270 ± 23718	49399 ± 18460	6.74E-02

TNF α	pg/mL	4.5 ± 2.3	4.3 ± 1.9	4.45E-01	8 ± 5.8	5.9 ± 3.4	1.02E-01
IFNr1	pg/mL	53.2 ± 56.6	53.7 ± 54	6.88E-01	30.9 ± 11.6	33.7 ± 9.7	8.31E-01
IL6	pg/mL	3.2 ± 5.5	1.9 ± 2.1	4.15E-02	4.6 ± 4.8	1.6 ± 1.4	3.91E-03
IL8	ng/mL	19.3 ± 30.8	12.9 ± 19	1.46E-01	30.2 ± 33.2	7.8 ± 11.1	8.30E-02
FGF19	MFI	23.7 ± 3.9	21.2 ± 5.2	1.43E-02	24.2 ± 3.9	21.9 ± 3.7	4.92E-02
FGF21	MFI	133.6 ± 56.5	132.1 ± 72.5	7.93E-01	182 ± 190	116.9 ± 85.7	2.59E-02
FGF23	MFI	47 ± 11.5	44.9 ± 15.5	4.87E-02	41.4 ± 7.2	37.8 ± 7.1	6.15E-02
CA	nmol/L	126±274.9	130.2±226.6	0.444234281	156±259	86.9±86.9	0.965820313
CDCA	nmol/L	322.8±446	455.6±1000.9	0.900083351	196.6±184.7	220.7±162.4	0.637695313
GCA	nmol/L	417.5±873.6	244±404.3	0.052696118	353.9±453.1	574.9±1001	0.637695313
GDCA	nmol/L	418.4±931.8	313.6±415.5	0.363786061	616.6±856.2	735.2±841.1	0.206054688
TCA	nmol/L	41.4±87.1	27.3±67	0.255575756	14.5±20.1	45.5±82	0.1015625
TDCA	nmol/L	31.4±59.4	23.4±27.1	0.594814425	22.6±18.6	52.7±58.2	0.1015625
TLCA	nmol/L	7.6±12.7	5.3±4.7	0.839255622	6.4±4.7	10.8±10	0.278320313
TUDCA	nmol/L	17.9±28.4	10.1±13.4	0.098183046	4.3±3.7	8.6±12.3	0.46484375
TaurinBA	nmol/L	203.1±327.4	137.2±194.2	0.151658527	105.3±80.9	258.2±346.8	0.067382813

MFI, mean fluorescence intensity determined by using the Luminex 200™ instrument. *, the unit of insulin is converted to uIU/L

Supplementary Methods

Screening, recruitment, dietary information and follow-up

171 Han Chinese patients with T2D were screened at baseline. The detailed inclusion and exclusion criteria are listed in **Supplementary Table 1**. Briefly, the eligibility criteria were as follows: 1) newly onset, treatment-naïve T2D patients diagnosed according to the 1999 World Health Organization criteria with fasting plasma glucose ≥7 mmol/L and/or an oral glucose tolerance test (OGTT) 2 h ≥11.1 mmol/L; 2) fasting glucose levels <13.0 mmol/L and HbA1c ≤ 10%; and 3) BMI ≤35 kg/m^2. The exclusion criteria were: 1) severe digestive diseases that might cause severe constipation or chronic or acute diarrhoea; 2) gastroenterological or other abdominal surgeries such as cholecystectomy within one year; 3) severe hepatic and renal failure (ALT>=2.5-fold above the normal

range and Scr>=132 μmol/L); 4) psychiatric disorders, severe infectious diseases, severe anaemia, or severe neutropenia; 5) severe cardiac diseases or cardiac function evaluation >=NYHA Class III; 6) acute diabetic ketonuria acidosis and hyperosmotic coma within 3 months; 7) past history of antidiabetic drug administration; and 8) treatment with antibiotics during the past 3 months.

Acarbose (Bayer HealthCare, Wuppertal, Germany) or Glipizide (Sine Pharm. Corp, Shanghai, China) was administered after a one-day run-in preparation. All the enrolled patients completed a physical examination and questionnaires for diet, lifestyle and history of disease and medication use. For Acarbose, the initial dose was 75 mg/day, 25 mg per meal, 3 times a day and, and the dose was increased to 300-450 mg/day, 100-150 mg per meal, 3 times a day within one month. For Glipizide, the initial dose was 5 mg/day, 2.5 mg pre-meal, twice a day, and the dose was increased to 15 mg/day, 5 mg pre-meal, 3 times per day. Patients were instructed to avoid ingesting yogurt and antibiotics or to keep record of prescriptions if necessary during the follow-up.

Dietary intake was recorded by a food frequency query (FFQ) based on the SLAN study[1], using a Chinese translated version. Primarily, the distribution of vegetarian or non-vegetarian and diary-food consumer or non-dairy-food consumer did not differ between the two arms at baseline (Pearson's Chi-squared test, $P>0.05$). The frequencies of vegetable, meat, fish, sweets intakes, and the frequencies of eating out were similar in patients belonging to the two treatment arms pre- and post-treatment (Kruskal-Wallis rank sum test, $P>0.05$). These dietary features of patients in both arms showed no significant changes after treatment (Kruskal-Wallis rank sum test, $P>0.05$).

Functional annotation of genes involved in BA synthesis based on BLASTP and BlastKOALA

To identify genes involved in bile acid metabolism, we performed a BlastP search against 1,011 amino acid sequences annotated as enzymes involved in BA metabolism in the UniProt database using an E value＜1E-5 [2] and bit score＞60 [3,4]. This resulted in 16,102 genes potentially involved in bile acid metabolism. We next assessed the 16,102 potential hits against KEGG using the KEGG online BlastKOALA algorithm[5]. As shown in **Supplementary Data 4**, the genes annotated to *bsh*, *baiE* and *baiI* by our pipeline showed high consistency with the KEGG online results, with 69%-85% genes annotated to the same KO. For genes annotated to *baiG* (bile acid transporter), 72.2% were assigned to K03453 (bile acid:Na+ symporter, BASS family), although no KO was reported and assigned to *baiG*. For genes annotated to *7β-hsdh* by our pipeline, 92.3% were assigned to K07124. By contrast, the remaining UniProt annotated genes exhibited rather low consistency with the KEGG online results. For example, genes annotated to *baiF* (K15871, EC 3.1.2.26, bile acid-CoA hydrolase) by UniProt were largely assigned to K18702 (uctC; CoA:oxalate CoA-transferase [EC:2.8.3.19], 43.4%), K07749 (frc; formyl-CoA transferase [EC:2.8.3.16], 31.0%) and K08298 (caiB; L-carnitine CoA-transferase [EC:2.8.3.21], 13.3%) by KEGG. For genes annotated to *baiA* (K15869, EC 1.1.1.395, 3α-hydroxy bile acid-CoA-ester 3-dehydrogenase) by UniProt, more than 80% of the genes were assigned to K00059 (abG; 3-oxoacyl-[acyl-carrier protein] reductase [EC:1.1.1.100]) by KEGG. There are several possibilities explaining the inconsistency between the two methods. First, the high sequence identity between target KOs (enzymes) and other KOs (enzymes). For example, the KEGG annotated CoA-transferases exhibit very high identity with bile acid-CoA hydrolase. The protein sequence A0A0S2W5Y8 (EC2.8.3.16, K07749) exhibits 99.7% identity with sequence A0A1C6GV35 (EC 3.1.2.26, bile acid-CoA hydrolase); the sequence F7UY98 (K08298) exhibits 92.3%

identity with sequence T4BVW9 (EC 3.1.2.26, bile acid-CoA hydrolase). Second, there are differences between the two annotation algorithms. For example, the gene encoding protein sequence 1 (ID: V1.CD3-0-PN_GL0121220) is annotated to *baiA* ((K15869) by UniProt but assigned to K00059 by KEGG, although protein sequence 1 exhibits higher identity (87%) with ag:ACF20977 (K15869) than with sequences from K00059 (**Supplementary Data 5**). Finally, the inconsistency could be also caused by unique protein sequences in the two databases. For instance, 12 of 13 genes annotated to *7β-hsdh* (7-beta-hydroxysteroid dehydrogenase, EC 1.1.1.201) are assigned to K07124 by KEGG. The protein sequences encoded by these genes exhibit more than 70% identity with R9UAM1 (7-beta-hydroxysteroid dehydrogenase, EC 1.1.1.201), and less than 40% identity with sequences from K07124 (**Supplementary Data 6**).

To further validate the annotation of genes involved in bile acid metabolism, we searched the 16,102 genes against the entire UniProt database (Release 2017_07 of 05-Jul-2017) and calculated the percentage of genes with the best-scoring hits assigned to the same BA metabolism functions/enzymes identified in the initial search against the 1,011 amino acid sequences annotated as enzymes involved in BA metabolism in the UniProt database (**Supplementary Data 7**). Due to the multiple submitted names of the same gene or protein and the existence of a massive number of uncharacterized proteins in UniProt, we combined the different synonymous protein names for each BA metabolism function and removed the best scoring uncharacterized proteins.

For *bsh*, *baiE*, *baiG*, *baiI* and *7β-hsdh* genes, the annotation of the highest scoring UniProt hits representing more than 85% of the genes was consistent with the annotations by searching against only the 1,011 amino acid sequences from UniProt database. These results

demonstrated the robustness of the annotation of these genes. However, for *baiF*, *baiCD*, *baiB*, *baiH*, *baiA* and *7α-hsdh*, their highest scoring UniProt hits were still largely assigned to non-BA metabolism functions.

Thus, although E value cutoff (E value＞1E -5) [2] and bit score cutoff (Score＞60) [3, 4] have been widely used for metagenomic functional annotation in previously published studies, our analyses clearly reveal the limitations to distinguish different enzymes/functions with high sequence identity. Accordingly, in the present study only genes exhibiting highly consistent annotation (*bsh*, *baiE*, *baiG*, *baiI* and *7β-hsdh*) have been included in the analyses.

Supplementary References

1. Harrington J, Fitzgerald AP, Layte R, Lutomski J, Molcho M, Perry IJ. Sociodemographic, health and lifestyle predictors of poor diets. *Public Health Nutr* **14**, 2166-2175 (2011).

2. Tremaroli V*, et al.* Roux-en-Y Gastric Bypass and Vertical Banded Gastroplasty Induce Long-Term Changes on the Human Gut Microbiome Contributing to Fat Mass Regulation. *Cell metabolism* **22**, 228-238 (2015).

3. Harrington ED*, et al.* Quantitative assessment of protein function prediction from metagenomics shotgun sequences. *Proceedings of the National Academy of Sciences of the United States of America* **104**, 13913-13918 (2007).

4. Tringe SG*, et al.* Comparative metagenomics of microbial communities. *Science* **308**, 554-557 (2005).

5. Kanehisa M, Sato Y, Morishima K. BlastKOALA and GhostKOALA: KEGG Tools for Functional Characterization of Genome and Metagenome Sequences. *J Mol Biol* **428**, 726-731 (2016).
