# Supplementary material for: Analyses of gut microbiota and plasma bile acids enable stratification of patients for antidiabetic treatment
Source: Nat Commun. 2017 Nov 27;8:1785. doi: 10.1038/s41467-017-01682-2 (PMC5702614; doi:10.1038/s41467-017-01682-2)
Supplement: Supplementary file 3 — Description of Additional Supplementary Files [file 41467_2017_1682_MOESM3_ESM.pdf]

Description of Additional Supplementary Files

File Name: Supplementary Data 1
Description: Generalized estimated equations analysis of correlations between changes in mOTUs and BA compositions induced by Acarbose treatment, adjusted for BMI, sex and age

File Name: Supplementary Data 2
Description: The taxa distributions of genes involved in bile acid metabolism

File Name: Supplementary Data 3
Description: Bacterial pathways and modules responsive to treatment with Acarbose and Glipizide (Reporter Score >1.95)

File Name: Supplementary Data 4
Description: Comparison of bile acid metabolism gene annotation based on BLASTP and BlastKOALA

File Name: Supplementary Data 5
Description: Comparison of annotation results of gene encoding V1.CD3-0-PN_GL0121220 based on BLASTP and BlastKOALA

File Name: Supplementary Data 6
Description: Comparison of annotation results of gene encoding 7β-HSDH based on BLASTP and BlastKOALA

File Name: Supplementary Data 7
Description: Validation of bile acid metabolism gene annotation by BlastP against the entire UniProt database
